# An innovative *bis*-allyl rhodanine robust red fluorophore for wide-range optical pH sensing with reversible and durable acidic–alkaline OFF/ON fluorescence

**DOI:** 10.1038/s41598-026-51070-4

**Published:** 2026-07-10

**Authors:** Wael A. A. Arafa, AbdElAziz A. Nayl, Ahmed I. Abd-Elhamid, Ismail M. Ahmed, Abd El-Naby I. Essawy, Stefan Bräse, Amr A. Essawy

**Affiliations:** 1 Department of Chemistry, College of Science, Jouf University, Sakaka, 72341 Al Jouf Kingdom of Saudi Arabia; 2 Composites and Nanostructured Materials Research Department, Advanced Technology and New, Materials Research Institute, City of Scientific Research and Technological Applications (SRTA-City), New Borg Al-Arab, Alexandria, 21934 Egypt; 3 Department of Chemistry, Faculty of Science, Fayoum University, Fayoum, 63514 Egypt; 4 Institute of Biological and Chemical Systems-Functional Molecular Systems (IBCS-FMS), Kaiserstrasse 12, 76131 Karlsruhe, Germany

**Keywords:** *Bis*-rhodanine, Fluorophore, Spectrofluorometric pH sensor, Donor–π–acceptor system, Reversible fluorescence OFF/ON, Red emission, Chemistry, Environmental sciences

## Abstract

A sustainable, ultrasound-assisted approach for synthesizing a *bis*-rhodanine derivative (BR) was established using *N*-allylrhodanine and 5-bromo-2-hydroxyisophthalaldehyde, employing a recyclable glycerol/proline (2:1) deep eutectic solvent. Structural characterization was unequivocally confirmed by NMR, FT-IR, HRMS, and elemental analysis. The red donor–π–acceptor fluorophore (BR) was evaluated as a wide-range spectrofluorometric pH probe. At 5.0 µM, BR exhibits dual absorption maxima at 357 and 568 nm with high molar absorptivity, and a red emission band centered at 718 nm with a 149 nm Stokes shift. Fluorescence is reversibly governed by a protonation/deprotonation equilibrium: acidification (pH 6.5–1.0) quenches emission with strong linearity (*r* ≈ 0.998), whereas alkalinity (pH 8.5–12.0) stabilizes an ICT-favored emissive state and linearly sensitizes fluorescence (*r* ≈ 0.995). The signal stabilizes within ~ 30 s, remains durable under reversible cycling between the fluorescence OFF mode at pH 2.0 and the fluorescence ON mode at pH 10.5. Therefore, BR interestingly functions as a dual-regime fluorescent pH probe, exhibiting acid-induced quenching at low pH and base-induced sensitization at high pH, with an explicitly characterized near-neutral transition (pH 6.5–8.5) that bridges the two operating domains. Moreover, BR is highly selective to pH as it resists common ionic interferents. Finally, the developed BR-derived spectrofluorometric pH probe is validated for the assay of acidic and alkaline pH in real samples of tap and lake waters, as well as in fresh lemon and orange juices, with high accuracy (98.9–102.3% recovery) and precision (% RSD: 0.93–1.13%).

**Supplementary Information:**

The online version contains supplementary material available at 10.1038/s41598-026-51070-4.

## Introduction

pH, while simple in definition, governs speciation, solubility, corrosion, adsorption, catalysis, transport phenomena, and reaction kinetics through proton activity. In environmental waters, it controls metal hydrolysis/mobility, carbonate equilibria, and organic-matter charge, thereby influencing contaminant fate and treatment performance. In industry, pH dictates efficiency in dyeing/finishing, electroplating, etching, CIP cleaning, and alkaline processing, with deviations causing yield losses and equipment damage. Because many modern applications require continuous monitoring in small volumes, harsh media, or confined geometries, conventional glass electrodes, while remaining standard, can drift or foul and become unreliable in microvolumes, viscous media, and extreme pH conditions. These constraints have accelerated interest in optical pH sensing, where proton-dependent spectral changes enable remote readout, miniaturization, and compatibility with electromagnetically noisy or spatially restricted environments^1,2^.

Among optical strategies, fluorescence is especially powerful because it converts a small chemical perturbation, such as the transfer of a single proton, into a large photophysical signal. Recent reviews emphasize that fluorescence pH probes can be engineered for imaging compatibility and integration into portable platforms. Still, they also highlight a persistent challenge: maintaining the probing accuracy under extreme pH conditions without photobleaching, hysteresis, indicator leaching, or matrix sensitivity^1^. Mechanistically, pH responsiveness is typically realized by controlling excited-state deactivation pathways: intramolecular charge transfer (ICT), photoinduced electron transfer (PET), excited-state proton transfer (ESPT), or twisted intramolecular charge transfer (TICT), so that protonation/deprotonation toggles the balance between radiative emission and non-radiative decay. Time-resolved mechanistic studies of widely used intracellular probes illustrate how proton activity can reshape excited-state dynamics, clarifying why some fluorophores exhibit clean OFF/ON transitions. In contrast, others drift or yield ambiguous intermediate states^3^. In parallel, general design strategies have emerged for “zero-background” turn-on sensing, where protonation deliberately triggers or suppresses TICT-type non-radiative relaxation, yielding high contrast and improved analytical robustness^4^.

A particularly demanding frontier is the alkaline region. Many classical indicators and popular fluorescent scaffolds were optimized around neutrality or mild acidity; at high pH, some suffer hydrolysis, oxidative degradation, or aggregation changes driven by ionic strength and deprotonation. Consequently, the reported probes above pH ≈ 10 are comparatively scarce, despite the demand in industrial cleaning streams, concrete chemistry, alkaline wastewaters, and process control^5–7^. Recent alkaline-focused probes demonstrate feasibility but also underscore the need for improved durability and calibration simplicity. For example, *bis*(2-hydroxyphenyl)benzazole-derived probes exhibit turn-on behavior with high pK_a_ values and good reversibility in basic media, yet their usable duration and performance can remain structural- and medium-dependent^5^. Perylene *tetra*-(alkoxycarbonyl) derivatives have been adapted as alkaline probes with practical strip-format potential, showing that rigid π-systems can be tuned to translate deprotonation into measurable fluorescence changes^7^. Coumarin-based designs have also been tailored for highly alkaline pH, including polymerizable indicators that yield photostable responses across the basic range^6^. More recently, BODIPY-based platforms have been reported for naked-eye/fluorometric recognition of highly alkaline pH, reflecting ongoing efforts to combine chemical robustness with strong optical contrast^8^. Among the most successful molecular frameworks employed in chemosensor design, rhodanine, a five-membered heterocyclic scaffold, has emerged as a highly effective sensing unit^9–11^. Owing to the presence of thiocarbonyl and carbonyl sites within a heteroatom-rich framework, rhodanine-based scaffolds demonstrate pronounced electronic modulation and coordination behavior, thereby enabling sensitive signal transduction upon external stimulation^12,13^. Compared with conventional fluorophores, rhodanines have advantageous photophysical properties, including high environmental sensitivity (pH, solvent effects, and polarity), outstanding photostability, structural flexibility, and strong fluorescence emission, enabling rational optimization of sensing performance. For example, a rhodanine-cyanine conjugate probe has been designed as a hybrid spectroscopic and chromogenic system for pH measurement over a wide pH range (1.0–14.0), with unique spectral and colorimetric transitions^14^. Also, a rhodanine-rhodamine-based fluorescent probe (pKa = 4.85) has been reported to have remarkably sensitive and well-defined pH-dependent fluorescence responses. It behaves linearly within a small pH range of 4.2–5.2, allowing for precise detection of slight proton shifts in moderately acidic conditions^15^. Beyond sensing applications, rhodanine-based compounds have shown notable biological activities, including anticancer, antiviral, and antibacterial activities^16–18^. These characteristics, along with their chemical robustness, low toxicity, and biocompatibility, improve their stability for integration into biomedical and biological sensing systems. Consequently, rhodanine-derived fluorescent chemosensors have been effectively used in various fields, including industrial process monitoring, food safety control, biomedical diagnostics, and environmental monitoring. Crucially, systematic modification of the rhodanine framework facilitates the design of sensors exhibiting superior selectivity, sensitivity, operational reliability, and reproducibility for targeted analytical applications^19^. Nonetheless, the majority of previously documented rhodanine-based frameworks originate from mono-substituted or structurally simplistic scaffolds that inherently restrict the regulation of intramolecular charge transfer (ICT) processes. This limitation hinders the precise adjustment of photophysical properties and pH-dependent optical responses, which are generally restricted to narrow acidic or near-neutral ranges^9,14,15^. Addressing these limitations necessitates the design of structurally superior rhodanine platforms that exhibit improved electronic delocalization and adjustable ICT properties to attain superior and more resilient sensing performance. In the present study, these challenges are addressed by designing a *bis*-allyl rhodanine-based system with a dual-acceptor architecture.

In this work, we introduce (5*Z*,5’*Z*)-5,5’-((5-bromo-2-hydroxy-1,3-phenylene)*bis*(methanylylidene))*bis*(3-allyl-2-thioxothiazolidin-4-one) (BR), a new donor–π–acceptor red fluorophore engineered as a dual-regime pH optical sensor with fluorescence “OFF” behavior upon progressive acidification (pH 6.5→1.0) and fluorescence “ON” sensitization under alkalinity (pH 8.5→12). The molecular design couples a phenolic core as a classical proton-responsive component with conjugated two rhodanine-like acceptor units to amplify ICT and to create distinct protonation states with measurably different radiative/non-radiative balances suiting an approach aligned with contemporary TICT/ICT gating principles. Probes with large Stokes shifts and stabilized ICT behavior are increasingly appreciated because they mitigate self-absorption and inner-filter distortions, improve signal discrimination, and simplify instrument settings—especially in concentrated or optically dense media^20^. The probe targets a wide pH range with a fast response while preserving linear calibration segments and OFF/ON reversibility under repeated cycling, thereby addressing the scarcity of sensors that remain quantitative, durable, and photophysically well-behaved at both ends of the pH spectrum, thereby enabling transferability to real sensing formats^1,2,21^.

## Experimental

### Materials

All reagents met analytical-grade specifications and were employed without additional purification unless stated otherwise. Bi-distilled water was used for all general solution preparations. Spectroscopy-grade methanol was obtained from Sigma-Aldrich and used directly. Pure grade salts: Zn(NO_3_)_2_, Cu(NO_3_)_2_, Co(NO_3_)_2_, Fe(NO_3_)_3_, Mg(NO_3_)_2_, Mn(NO_3_)_2,_ Hg(NO_3_)_2_, Al(NO_3_)_3_, Cd(NO_3_)_2_, NaCl, KCl and CaCl_2_ were the sources for metal cations and NaNO_2_, NaNO_3_, Na_2_SO_4_, Na_2_CO_3_, NaHCO_3_, KBr, KI and K_3_PO_4_ salts were the sources for anions in addition to NaCl used also as source of chloride were obtained from Aldrich. Hydrochloric acid and sodium hydroxide of analytical grade, as well as organic reagents used to prepare *bis*-rhodanine, were obtained from Sigma-Aldrich.

### Methods

####  Synthesis of 5-bromo-2-hydroxyisophthalaldehyde (2)^22^

A mixture of 4-bromophenol (**1**, 1.0 mmol) and hexamethylenetetramine (8.0 mmol) in anhydrous trifluoroacetic acid (25.0 mL) was heated at 110 °C for 48 h, affording a yellow solution. Upon cooling to room temperature, the reaction mixture was added to an aqueous HCl solution (4 M, 50.0 mL) and stirred for 5 h. The precipitated solid was collected by filtration and washing with water (3 × 15.0 mL) to yield the desired product as yellow crystals (93%).

#### Synthesis of (5*Z*,5’*Z*)-5,5’-((5-bromo-2-hydroxy-1,3-phenylene)*bis*(methanylylidene))*bis*(3-allyl-2-thioxothiazolidin-4-one) (4)

A mixture of 5-bromo-2-hydroxyisophthalaldehyde (2, 1.0 mmol) and 3-allyl-2-thioxothiazolidin-4-one (3, 2.0 mmol) in glycerol/proline (2:1, 5.0 g) was sonicated at 80 °C for 90 min as monitored by TLC. Upon addition of water (20.0 mL), a solid precipitated and was collected by filtration, then washed with ethanol (3 × 10.0 mL) to afford compound 4 in 96% yield without further purification (See SI).

#### Preparation of the working solutions and measurement methodology

A 5.0 × 10^−3^ mol L^−1^ stock solution of BR probe was prepared by dissolving 67.4 mg in 3.0 mL DMF, then completed with methanol to 25.0 mL in a calibrated measuring flask. After that, a working solution 5.0 × 10^− 6^ mol. L^−1^ from BR was prepared by pipetting 10.0 µL of the stock solution, followed by diluting with deionized water in a 10.0 mL measuring flask. To adjust the pH, very small volumes of hydrochloric acid and sodium hydroxide were used. The effect of the metal cations and anions on the fluorescence intensity was examined by adding portions of the cation stock solution to a known volume of the fluorophore solution (10.0 mL). The addition was limited to 100.0 µL to keep the dilution insignificant. The fluorescence intensity of the BR sensor (5.0 × 10^−6^ mol. L^−1^) was monitored, revealing an emission band at λ_em_ = 718 nm (slits, 5 nm) upon optimal excitation at λ_ex_ = 580 nm (slits, 5 nm). The effects of different acid or alkaline pH concentrations within the ranges (1.0–6.5) and (8.5–12.0) on the fluorescence intensity of the BR probe were recorded, where a calibration curve was developed.

#### Quantum yield estimation

The fluorescence quantum yield (Φ) quantifies the effectiveness of converting absorbed photons into radiative emission. As such, it provides a sensitive probe of excited-state relaxation pathways, including non-radiative deactivation processes, and it can also reflect sample purity and structural features of the fluorophore^23^. In this work, relative quantum yields were evaluated under identical instrumental and experimental conditions by benchmarking the integrated emission response of the investigated sample against a reference dye. Rhodamine B dissolved in ethanol, with a reported quantum yield of *Ф*_s_ = 0.65, was used as the calibration standard^24^. The quantum yield of the unknown system (*Ф*_u_) was obtained using:

1 $$\:\varnothing\:=\frac{{{\varnothing}}_{\mathrm{s}}{\mathrm{F}}_{\mathrm{u}}\:\left({OD}_{s}\right){n}_{u}^{2}}{{\mathrm{F}}_{\mathrm{s}}\left({\mathrm{O}\mathrm{D}}_{\mathrm{u}}\right){\mathrm{n}}_{\mathrm{s}}^{2}}$$

where F_u_ and F_s_​ denote the integrated fluorescence emission intensities of the unknown and standard, respectively; OD_s_ and OD_u_​ are the corresponding optical densities at the excitation wavelength; n_s_, and n_u_​ represent the refractive indices of the media for the unknown and the standard solutions.

#### Apparent pKa determination

To quantify the sensitivity midpoint of the BR probe, the pH-dependent fluorescence response was analyzed using a sigmoidal model. The integrated fluorescence intensity *F* was calculated at various pH values. The estimates were normalized and fitted to estimate the apparent *pK*_*a*_​.

#### Real sample analysis

Fresh lemon and orange juices were prepared, and real water samples (tap water and lake water) were collected and filtered through 0.45 μm membrane filters. The pH of each sample was measured using a conventional pH meter. Aliquots of BR stock solution were added to each sample to achieve a final concentration of 5.0 µM. The protocol for lemon and orange juices is to dilute 3.0 mL of fresh juice to 10.0 mL with deionized water. Fluorescence intensities were measured, and pH values were determined from the calibration curve. The accuracy and precision of the developed spectrofluorometric method were evaluated by comparison with electrode measurements.

### Instrumentation

UV–visible absorption spectra were recorded using an Agilent Cary 60 UV–Vis spectrophotometer, while steady-state photoluminescence data were acquired on a Cary Eclipse fluorescence spectrophotometer (Agilent Technologies, USA). All optical measurements were performed in 1.0 cm path-length quartz cuvettes, using 5 nm bandpass (slit width) settings for both excitation and emission. The pH of each solution was tracked using a Jenway 3040 ion analyzer. FTIR spectra were recorded on Shimadzu IR-Tracer 100 (Kyoto, Japan). ^1^H/^13^C NMR spectra were obtained on a Jeol 600 MHz spectrometer (JEOL, Peabody, MA, USA) in DMSO-*d*_*6*_ and acetone-*d*_*6*_.

## Results and discussion

### Synthesis and characterization of *bis*-rhodanine derivative (4)

**Scheme 1:**
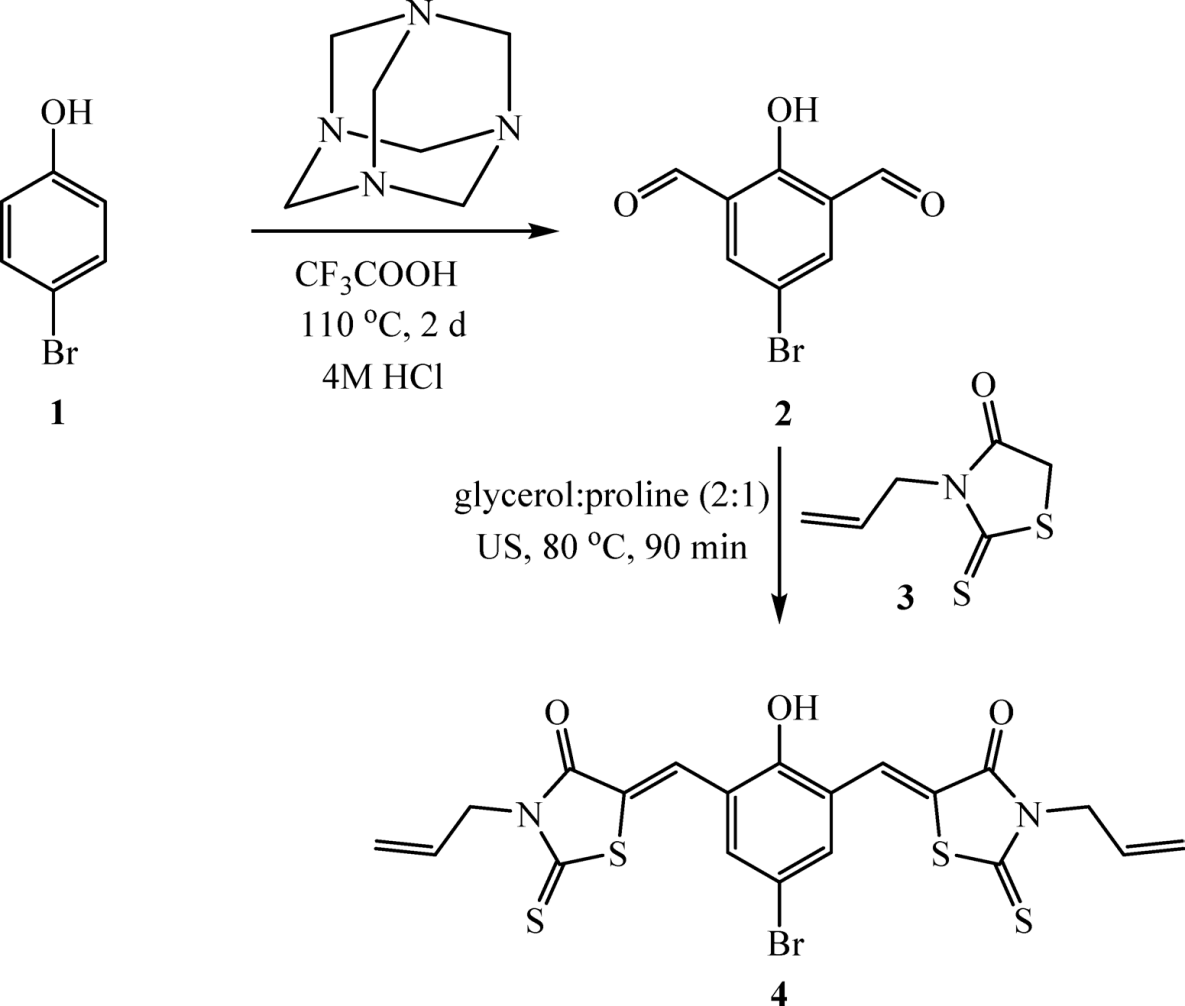
Ultrasound-assisted synthesis of *bis*-rhodanine (4).

As outlined in Scheme 1, 5-bromo-2-hydroxyisophthalaldehyde (2) was acquired *via* the Duff reaction of 4-bromophenol (1) with hexamethylenetetramine in trifluoroacetic acid^22^. The targeted *bis*-rhodanine derivative (4) was successfully obtained *via* the reaction of 5-bromo-2-hydroxyisophthalaldehyde (2) and *N*-allyl rhodanine (3) in glycerol: proline (2:1) as a deep eutectic solvent (dual role: catalyst and solvent) employing ultrasonic irradiation for 90 min at 80 °C. The reaction proceeded smoothly, resulting in a high yield (96%) of the anticipated *bis*-rhodanine (4). Herein, ultrasound offers several advantages over traditional methods, including accelerating reagent dispersion and interaction, optimizing reaction kinetics, improving mass transfer, and stimulating particle growth^25^. Moreover, the deep eutectic mixture of proline and glycerol has been shown to be an efficient, sustainable alternative to traditional solvents and catalysts. The suggested chemical structure of *bis*-rhodanine (4) was verified employing various techniques, including NMR, FT-IR, HRMS, and elemental analyses. For instance, the ^1^H NMR spectra of *bis*-rhodanine (4) revealed two singlets attributed to the two vinylic protons (=CH–) at *δ* 8.21 ppm. The signals at δ 190.36 and 166.63 ppm have been assigned to C=S and C=O motifs, respectively, based on ^13^C NMR. As anticipated, the condensation reaction produced two potential stereoisomers, *E* and *Z*. The *Z*-isomer had been formed selectively over the *E*-isomer, distinguished by a downfield shift in methine-motif protons (=CH–). Furthermore, high-resolution mass spectrometry (HRMS, ESI, positive ion mode) validated the molecular formula of the *bis*-rhodanine derivative (4), showing a molecular ion peak consistent with the expected species. The calculated mass for C_20_H_15_BrN_2_O_3_S_4_ ([M]^+^) is 537.9149, whereas the experimentally obtained value is 537.9197, with a very small variance (Δ*m/z* = + 0.0048). This is within the permissible instrumental error range and validates the suggested structure. The sustainability of the developed protocol is underscored by its favorable green chemistry metrics. Notably, the high atom economy (AE = 93.8%), low process mass intensity (PMI = 1.11), and minimal environmental factor (E-factor = 0.11) demonstrate efficient material utilization and low waste generation. In the production of *bis*-rhodanine (4), the glycerol/proline (2:1) DES’s recyclability was investigated. After the process was finished, water was added to precipitate the product (4), which was then filtered out, and the DES was isolated from the filtrate by evaporating water at a lower pressure. The recovered DES was directly reused across four cycles, proving its stability and reusability with just a little yield drop (96% to 91%) and no discernible change in reaction time or product purity (Fig. S6).

### UV-vis. absorption and emission characteristics of the BR probe

**Fig. 1:**
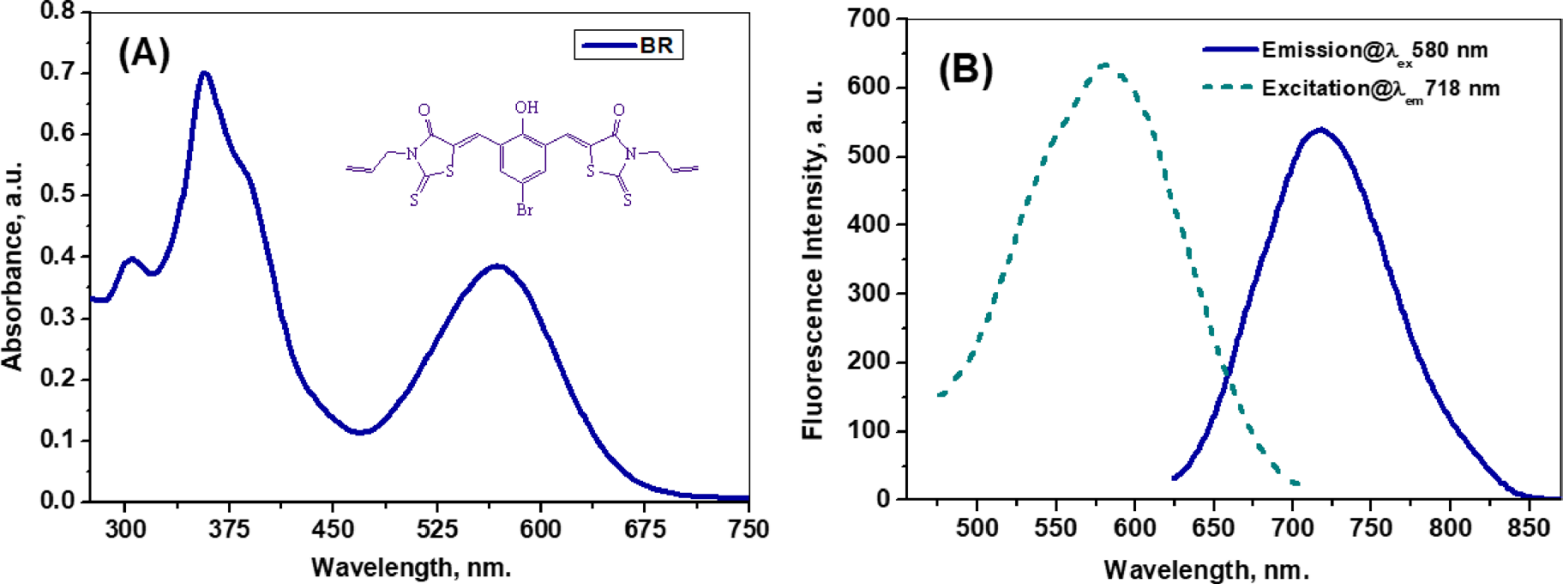
Absorption spectrum (**A**); Excitation and emission spectra (**B**) of the BR pH sensing fluorophore ([BR] = 5.0 µM).

The fundamental photophysical properties of the newly synthesized BR fluorophore (5.0 × 10^−6^ mol. L^−1^), which are paramount to its function as a pH optical sensor, are depicted in Fig. 1. In Fig. 1A, the absorption spectrum shows a strong high-energy band in the near-UV/blue region, with maximal absorbance at 357 nm, and a pronounced visible band centered at 568 nm. For a donor–π–acceptor chromophore of this type, this dual-band pattern is typically interpreted as the coexistence of a more localized π–π* transition (dominant at higher energy) of molar absorptivity (ε) 140,000 L mol^−1^ cm^−1^ facilitated by the extensive conjugated system within the fluorophore’s architecture and a proposed lower-energy ICT transition of ε = 77,000 L mol^−1^ cm^−1^, that becomes allowed when electron donation from the phenolic core couples efficiently through the conjugated linkers into the heteroatom-rich rhodanine acceptor termini. The fact that the visible absorption is broad and intense is consistent with an ICT-active scaffold and with the “push–pull” strategy, widely used to increase fluorophores’ responsiveness to their environments^4,26^. Importantly, the spectrum was recorded at (µM) concentration, which is typically chosen to minimize aggregation-driven spectral artifacts and directs the working spectral features to the intrinsic molecular electronic structure of the developed BR probe. Figure 1B verifies that the fluorescence originates from the same chromophoric system responsible for the visible absorption. The excitation spectrum recorded at λ_em_ = 718 nm (dashed line) peaks clearly above the absorption maximum, consistent with an angle-dominant missive species. If significant impurities or competing emissive aggregates dominate, the excitation maximum often shifts or exhibits multiband structure. The emission band is displaced deep into the red/near-IR edge, with a maximum at λ_em_ = 718 nm, yielding a clearly marked Stokes shift of 149 nm. This degree of separation is not merely a superficial photophysical characteristic; it constitutes a tangible benefit for sensing. Intensity-based fluorescence measurements may be influenced by inner-filter effects arising from excitation attenuation and/or emission reabsorption in optically dense media. Accordingly, the working micromolar concentration of BR as well as the large Stokes shift and far-red emission, which reduces secondary inner-filter/self-absorption artifacts where the large Stokes shift strongly suppresses spectral crossover between excitation light and collected fluorescence, reduces self-reabsorption, and improves signal-to-background when the fluorophore is deployed in colored or scattering media (exactly the kind of environments where optical pH measurements are often preferred over fragile electrodes)^1,2,4^. Mechanistically, such a wide Stokes shift is commonly associated with substantial excited-state relaxation, frequently proposed to be driven by ICT in donor–π–acceptor architectures, which rhodanine-derived acceptor moieties can effectively enforce. After photoexcitation, the charge distribution reorganizes, the excited state becomes more polar (and often more stabilized), and emission occurs from this relaxed state at a much lower energy than the initial absorption transition^4,5,20,21^. Moreover, the quantum yield (*Ф*) of the deveopled BR fluorophore referenced to rhodamine B reveals an estimte of 0.24. The fact that the fluorophore emits in the red is also strategically beneficial for sensing, whereas wavelength-shifted emission generally experiences less interference from ubiquitous background fluorescence and is more tolerant of sample coloration, a point repeatedly emphasized in modern fluorescent probe design^2,4^. Moreover, the quasi-mirror-image relationship between the absorption and emission profiles suggests minimal structural reorganization of the fluorophore in its excited state, which is conducive to a high fluorescence quantum yield.

Importantly, studying the photostability of the developed BR probe is crucial to address the reliability and practicality aspects. Therefore, BR was subjected to continuous UV irradiation for 60 min, and its UV–Vis absorption spectra were monitored over time. The results depicted in Figure S7 (See SI) confirm that BR retains its characteristic spectral profile with slight intensity attenuation, demonstrating good photochemical stability. The absorbance decay obeys pseudo-first-order kinetics with a small apparent rate constant (k = 0.0011 min^−1^) and strong linearity (*r* = 0.97), supporting robust performance under prolonged illumination conditions relevant to fluorescence sensing.

### Influence of acidic and alkaline pHs on the UV-vis. absorption features of the BR probe

**Fig. 2:**
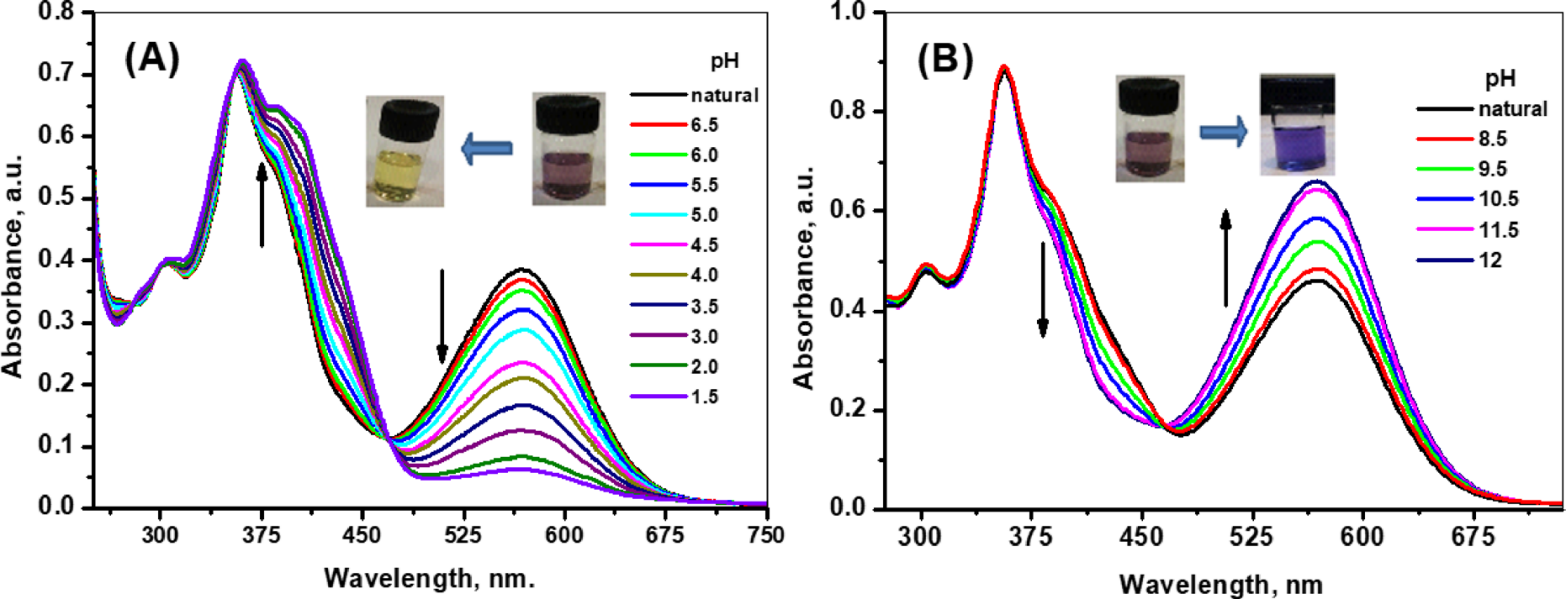
Variation of the absorption spectrum of the BR pH probe ([BR] = 5 µM) upon: increasing acidity from pH 6.5 to 1.5 (**A**); increasing alkalinity from pH 8.5 to 12.0 [Inset: images for the developed color changes].

Figure 2 shows that the developed fluorophore behaves as a true acid–base chromophore in the ground state, where proton activity reorganizes the electronic structure and, therefore, the absorption pattern. In Fig. 2A, progressive acidification from pH 6.5 down to pH 1.5 produces a systematic attenuation of the long-wavelength visible band. In contrast, the higher-energy band in the near-UV/blue region becomes comparatively more pronounced. This reciprocal evolution is the spectral signature expected when the ICT transition is gradually “switched off” by protonation. As the medium becomes more acidic, the donor–π–acceptor coupling is weakened (and/or the accepting heteroatom-rich fragment is proton-stabilized), reducing the oscillator strength of the low-energy ICT band and shifting the chromophore toward more localized π–π* absorption at shorter wavelengths. Conceptually, the dye is being pushed from a strongly polarized, push–pull electronic distribution toward a less charge-separated ground state, which diminishes the visible ICT absorption responsible for the darker coloration at higher pH. Interestingly, the isosbestic point indicates that the system is dominated by interconversion between two principal absorbing forms: the weakly donating “neutral/protonated” state and a more conjugated form near neutrality. Moreover, the isosbestic behavior indicates that the developed BR probe does not strongly fragment into multiple absorbing byproducts during acid titration; instead, it largely toggles between defined electronic states, as shown in the inset image. As the visible ICT band collapses under strong acidity, the solution color changes accordingly, consistent with reduced absorption in the green–orange part of the spectrum that normally drives purple/red appearance^27^. Figure 2B depicts the complementary alkaline chemistry from pH 8.5 to 12.0, where increasing alkalinity again intensifies the long-wavelength visible absorption band while diminishing the shorter-wavelength band. This is the pattern expected for phenolic dyes whose deprotonation generates a phenolate donor: the phenolate dramatically increases electron donation into the π-system, strengthens ICT, and stabilizes the low-energy transition, yielding the markedly larger molar absorptivity. In donor–acceptor chromophores, this deprotonation-driven enhancement of charge transfer is the same “push–pull amplification” frequently discussed across modern colorimetric/fluorometric sensor chemistry, because it turns a small chemical incident (loss of proton) into a large optical change seen in the growth of a strong visible band^28,29^. The inset images in Fig. 2B are consistent with this mechanism: the emergence and growth of the visible band produces a deeper purple/blue coloration at higher pH, enabling naked-eye discrimination. Contemplating the smooth, ordered changes in absorption features across both acidic and alkaline steps implies a predictable proton-coupled electronic response, which is crucial for durable optical pH sensing. Modern mechanistic work on widely used “OFF–ON” pH fluorophores underscores that reliable switching requires a well-defined coupling between the protonation state and the chromophore’s electronic structure, which is strongly emphasized for the developed BR probe at the ground-state absorption level^30^.

### Linearity and sensitivity of the BR probe in the fluorescence OFF/ON pH-titration in the acid and alkaline media

**Fig. 3:**
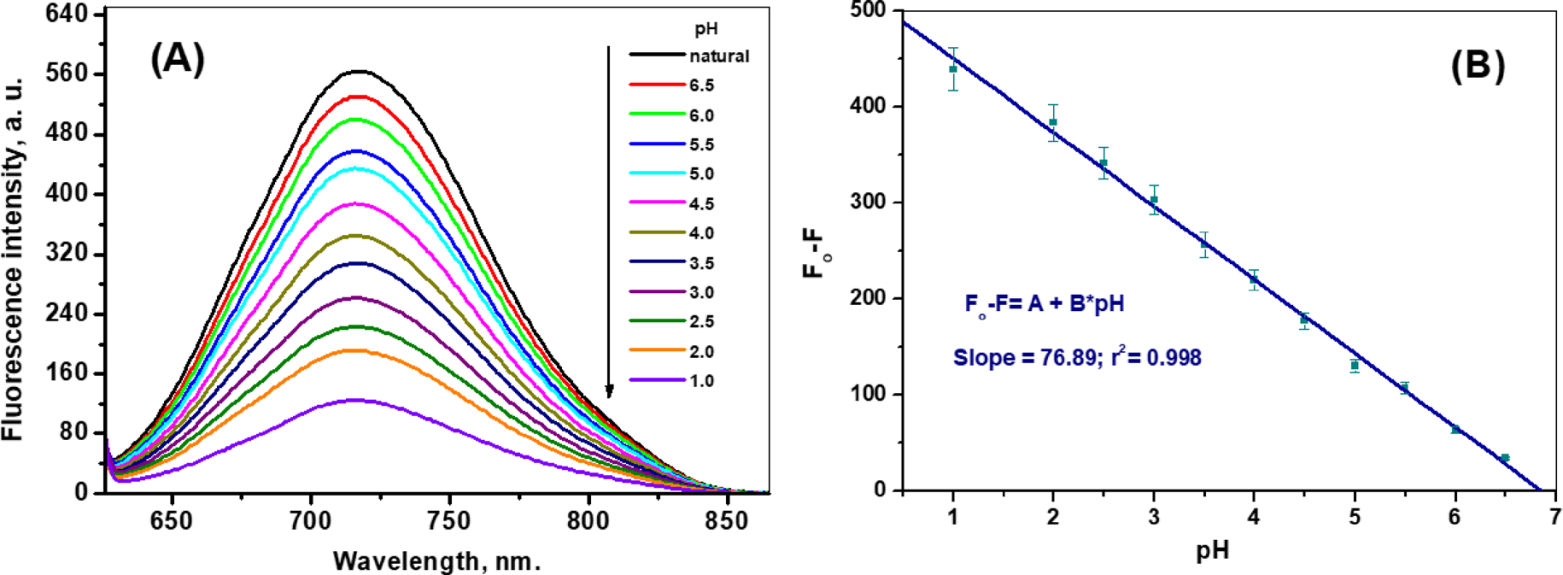
Variation of the fluorescence spectrum of the BR pH probe ([BR] = 5.0 µM) upon increasing acidity from pH 6.5 to 1.0. “Fluorescence quenching” (**A**); the linear regression equation and the developed calibration curve (**B**).

The developed fluorophore’s function as a highly sensitive acidic pH probe is clearly demonstrated in Fig. 3, which shows its dynamic fluorescence response to increasing proton concentration. In Fig. 3A, the shape of the BR emission band is conserved while the peak intensity decreases systematically as the solution is acidified from pH 6.5 down to pH 1.0. This pattern strongly suggests that the pH response is governed primarily by interconversion between closely related molecular states rather than by dye decomposition or the appearance of multiple emissive impurities. That distinction is crucial where intensity-based pH sensing only becomes reliable when the probe undergoes a controlled protonation equilibrium that changes the radiative/non-radiative balance without creating a moving target of new species^31,32^. The fluorescence quenching with increasing acidity is consistent with proton-controlled suppression of the proposed ICT emissive state. In donor–π–acceptor fluorophores, acidification can diminish effective donor strength and/or protonate heteroatom-rich acceptor regions, which redistributes electron density in the excited state and often opens faster non-radiative internal conversion and vibrational relaxation decay channels. Moreover, protonation can (i) weaken ICT oscillator strength, (ii) increase conformational freedom that promotes twisted charge-transfer relaxation, or (iii) enable PET-like quenching routes depending on how the frontier orbitals shift with protonation^33^.

The calibration curve in Fig. 3B plots F₀−F versus pH over the range 1–7, where F₀ is the reference fluorescence intensity, and F is the intensity at a given pH. The behavior is statistically linear, obeying the regression equation F₀ - F = A + B·pH, with a negative slope and a strong correlation (*r* = 0.998), meaning that each incremental increase in acidity produces a nearly proportional increase in quenching magnitude across the working range. Moreover, the analytical sensitivity of BR in probing acid media in terms of the limit of detection (LOD) was determined using the equation: LOD= 3*σ/S*, where *σ* is the standard deviation and *S* is the slope of the linear calibration plot. Accordingly, the estimated LOD in pH units is 0.21, reflecting the probe’s practical resolution. Furthermore, recovery percentages were calculated by the equation, Recovery (%) = (measured concentration/added concentration)×100 resulting in estimates within the range 99.2–100.05%, accompanied by %RSD ranging from 0.95 − 2.68%, revealing the high accuracy and precision of the developed BR probe in the fluorimetric assay of acidic pH.

**Fig. 4:**
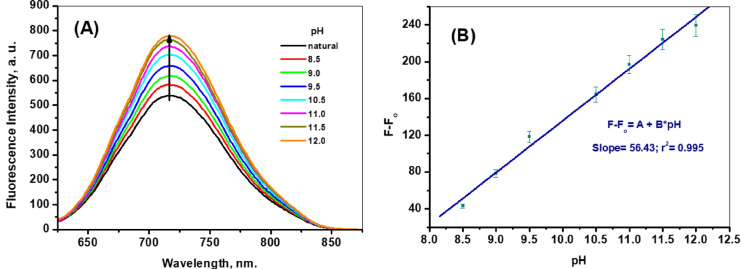
Variation of the fluorescence spectrum of the BR pH probe ([BR] = 5.0 µM) upon increasing alkalinity from pH 8.5 to 12.0. “Fluorescence sensitization” (**A**); the linear regression equation and the developed calibration curve (**B**).

Complementing its acidic response, the BR fluorophore exhibits a compelling, functionally opposite behavior in alkaline environments, as shown in Fig. 4. Figure 4A illustrates the phenomenon of “fluorescence sensitization,” in which a systematic, substantial, monotonic enhancement of emission intensity is observed as pH increases from 8.5 to 12.0. In contrast, the emission band retains essentially the same shape, with the emission maxima remaining centered at 718 nm and suggesting that the sensing event primarily modulates the radiative efficiency of a single dominant emissive state rather than generating a new emissive species. In molecular terms, this behavior is consistent with base-promoted deprotonation of the phenolic site (phenol⇌phenolate) and the accompanying redistribution of electron density across the extended π-system. For push–pull chromophores, converting a neutral phenol into a phenolate typically strengthens the donor character, amplifies ICT, and can either suppress or rebalance the non-radiative internal conversion or charge-transfer deactivation pathways, thereby increasing the output fluorescence intensity at higher pH^8,34^. Modern alkaline-range probe design often exploits a similar principle, engineering a high apparent pKa so that the deprotonated form becomes progressively populated only in basic media, which is why robust fluorescence reporters above pH ~ 9 remain comparatively rare and require careful control of electron-transfer and conformational-relaxation pathways^6,35^.

The pH-driven emission sensitization is quantitatively validated in Fig. 4B by the derived linear calibration curve. The fluorescence intensity shows a strong linear correlation with pH across the working alkaline range (8.5–12), described by the linear regression equation F-F₀ = A + B·pH, with a correlation coefficient *r* = 0.995. Interestingly, the LOD in pH units for the calibration in basic media reveals an estimates of 0.32. In addition, the estimated recovery percentages were 98.8–100.1%, with %RSD ranging from 0.53 to 1.84%, which confirms the high accuracy and precision of the developed BR probe in the fluorimetric assay of alkaline pH.

Because pH 6.5–8.5 is a biologically and environmentally important interval, the fluorescence behavior of BR was explicitly examined in this region by initiating the titration at pH 6.5 (slightly acidic) and then increasing alkalinity stepwise to pH 8.5. As shown in Figure S8A (See SI), the emission band centered at ~ 718 nm retains its overall shape while its intensity increases gradually with pH, evidencing a controlled sensitization rather than spectral distortion or the emergence of secondary emissive species. Quantitatively, the near-neutral response yields a robust calibration when expressed as ΔF = F−F_o_ (where F_o_ is the signal at pH 6.5): ΔF increases linearly with pH over 6.5–8.5 (r^2^ = 0.998) (Figure S8B; See SI). This demonstrates that BR pH probe remains responsive across the near-neutral interval, albeit with a smaller amplitude than in strongly alkaline media. Mechanistically, this behavior is consistent with the near-neutral regime being dominated by the neutral phenolic form, where only the onset of deprotonation occurs; consequently, ICT strengthening and radiative efficiency increase progressively but do not yet reach the fully deprotonated, strongly emissive phenolate-dominated state observed at higher pH.

Furthermore, while segmented linear regressions are useful for calibration within working pH range, a rigorous comparison of pH probes requires the apparent *pK*_*a*_. Therefore, the integrated fluorescence response of BR was fitted as a function of pH using a sigmoidal (Boltzmann/logistic) equation. Accordingly and utilizing the the integrated fluorescence estimates either due to the fluorescene OFF *via* acidification in addition to the fluorescence ON *via* alkalization within the pH 1–12, the deveopled BR probe exhibits an apparent acidity constant “*pK*_*a, app*_*”* ​≈ 5.2. This value is independently supported by a half-intensity interpolation, which places the midpoint at ≈ 5.30, confirming the reliability of the sigmoidal estimate.

### Response kinetics and photostability of BR fluorophore in pH-fluorimetric assaying

**Fig. 5:**
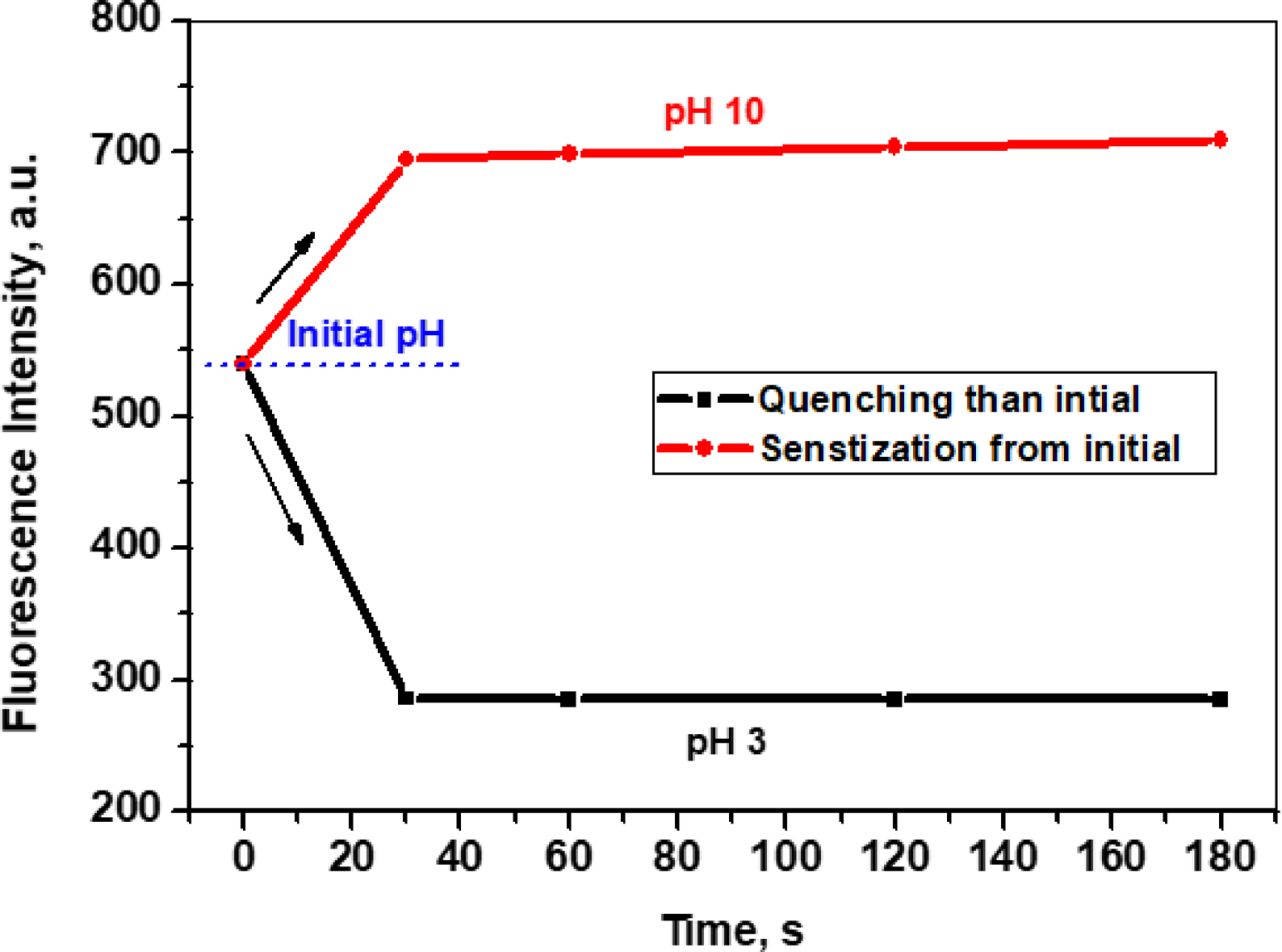
Response time and constancy of fluorescence intensities of the BR pH probe in cases of quenching due to acidification (pH 3.0) or sensitization due to alkalization (pH 10.0).

The practical utility and robustness of a fluorescent sensor are critically dependent not only on its spectral response but also on its response kinetics and operational stability, parameters which are rigorously evaluated in Fig. 5. This figure shows the real-time fluorescence dynamics of the developed fluorophore during rapid transitions between acidic (pH 3.0) and alkaline (pH 10.0) conditions. Starting from the initial neutral pH level at ≈ 5.3 × 10^2^ a.u., the response bifurcates depending on whether the medium is driven acidic or alkaline. Upon switching to an acidic condition, the fluorescence intensity drops sharply and reaches a stable low plateau near ≈ 2.8 × 10^2^ a.u. within the first 30 s, after which it remains essentially constant up to 180 s. The rapid approach to a flat baseline indicates that the OFF state refers to the chemically steady state of the developed BR fluorophore on the measurement timescale. This is the kinetic signature of a rapid fluorescence-quenching process dominated by proton-coupled electronic reorganization rather than by slow secondary processes such as aggregation, precipitation, or chemical degradation, in which acidification likely promotes a protonated form in which the emissive ICT state is suppressed and non-radiative relaxation becomes more competitive^36,37^. The alkaline switching behavior exhibits a turn-on jump from the initial intensity to a high plateau around 7 × 10^2^ a.u. over a similarly short response time, followed by a steady signal. This is the hallmark of fluorescence sensitization controlled by base-promoted deprotonation, most plausibly conversion of a phenolic donor into a phenolate, which strengthens the donor component of the push–pull system, amplifies ICT, and increases the radiative output without requiring a slow structural rearrangement^33,38^.

Accordingly, the developed BR fluorophore is not merely “pH sensitive” but also kinetically competent: it rapidly reaches its acid-quenched and base-sensitized states and then maintains them with high stability. This combination is especially valuable for wide pH-range operation because the extremes of the pH scale are where many indicators become unstable or slow due to competing chemistry. Such response kinetics and plateau stability are also consistent with recent reports on wide-range optical pH sensors^39,40^.

### Durability and pH-reversibility cycling of the developed BR fluorimetric probe

**Fig. 6:**
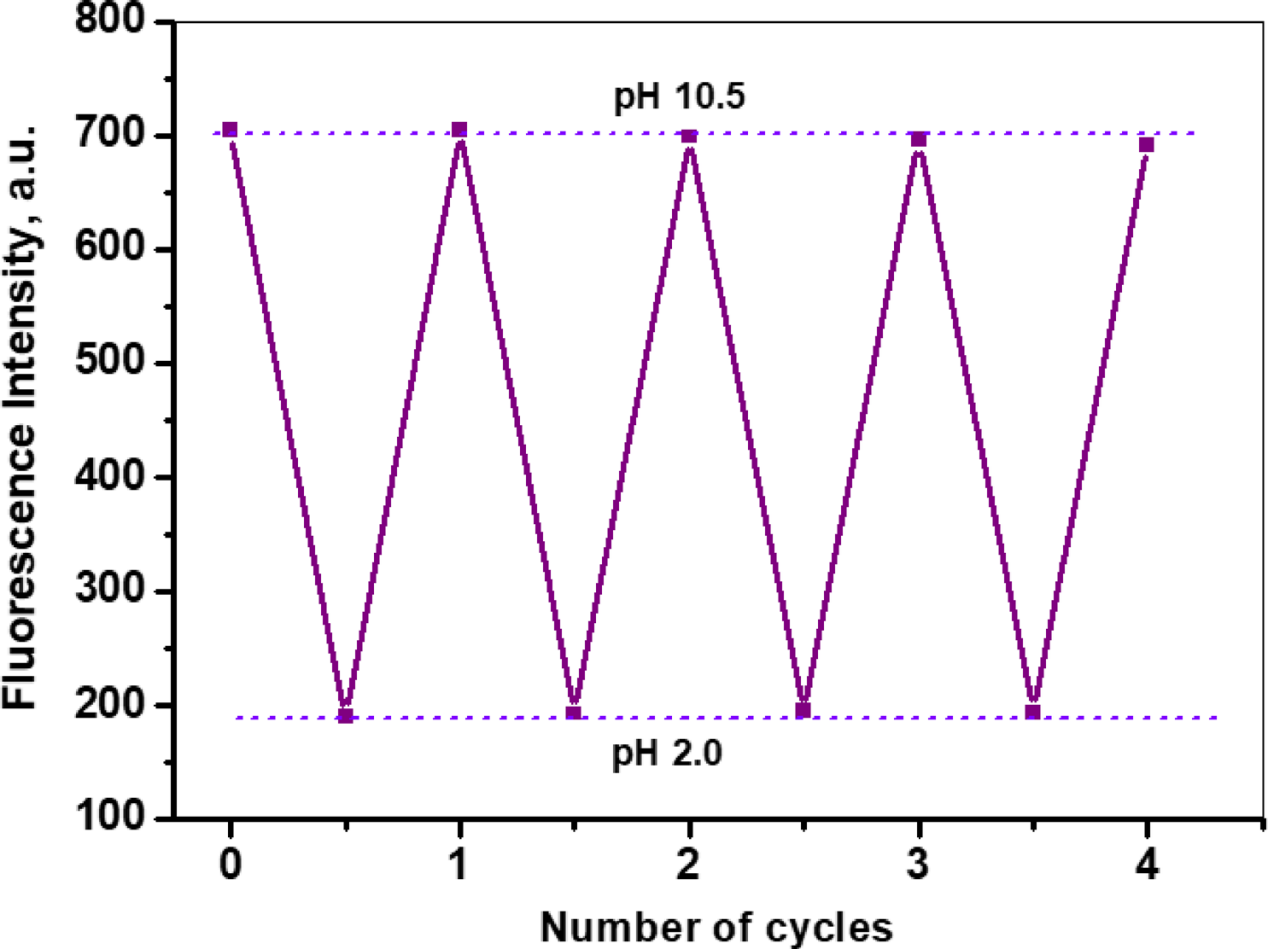
Durability study for the fluorescence OFF/ON reversibility of the BR pH probe at two tested pHs, 2.0 and 10.5.

The ultimate test for any functional sensor intended for practical, long-term deployment is its durability and cycling stability, a parameter critically assessed in Fig. 6. The plotted fluorescence intensity toggles reproducibly between two well-separated plateaus by repeatedly cycling the BR fluorophore between highly acidic pH 2.0 (Fluorescence OFF) and alkaline pH 10.5 (Fluorescence ON). In the alkaline state, the signal repeatedly returns to a high-intensity level of roughly ~ 7.0 × 10^2^ a.u., whereas in the acidic state it falls back to a low plateau near ~ 1.9 × 10^2^ a.u.; importantly, these values remain essentially unchanged from cycle to cycle over the full sequence (4 cycles). This near-constant amplitude and the absence of progressive signal loss indicate that the molecular structure remains largely intact, resisting the chemical degradation such as hydrolysis of the allyl or heterocyclic bonds, or photo-oxidation that often plagues organic fluorophores under such harsh and oscillating conditions; suggesting that the fluorescence modulation is governed primarily by a fully thermodynamically favored reversible protonation/deprotonation equilibrium at the phenolic and likely the rhodanine sites rather than by irreversible dye consumption, slow aggregation, or chemical degradation. This remarkable durability can be attributed to the intrinsic chemical stability of the molecular design^41,42^. The conjugated system, anchored by the robust 4-bromophenol core and stabilized by sulfur atoms in the rhodanine rings, appears to withstand repeated electronic and structural rearrangements associated with the switching process. In practical photophysics terms, the OFF/ON behavior is consistent with proton-controlled gating of an intramolecular charge-transfer emissive manifold: strong acid favors a protonated electronic structure with enhanced non-radiative decay (quenching), while strong base favors the deprotonated (phenolate-like) donor form that strengthens charge transfer and restores an emissive excited state (sensitization). Therefore, the developed BR fluorophore behaves as a genuinely reusable OFF/ON pH reporter across extreme proton activities, rather than a one-direction indicator whose signal drift would eventually corrupt calibration and practical deployment^42,43^.

### Effect of co-existing species on the selectivity of the BR probe

**Fig. 7:**
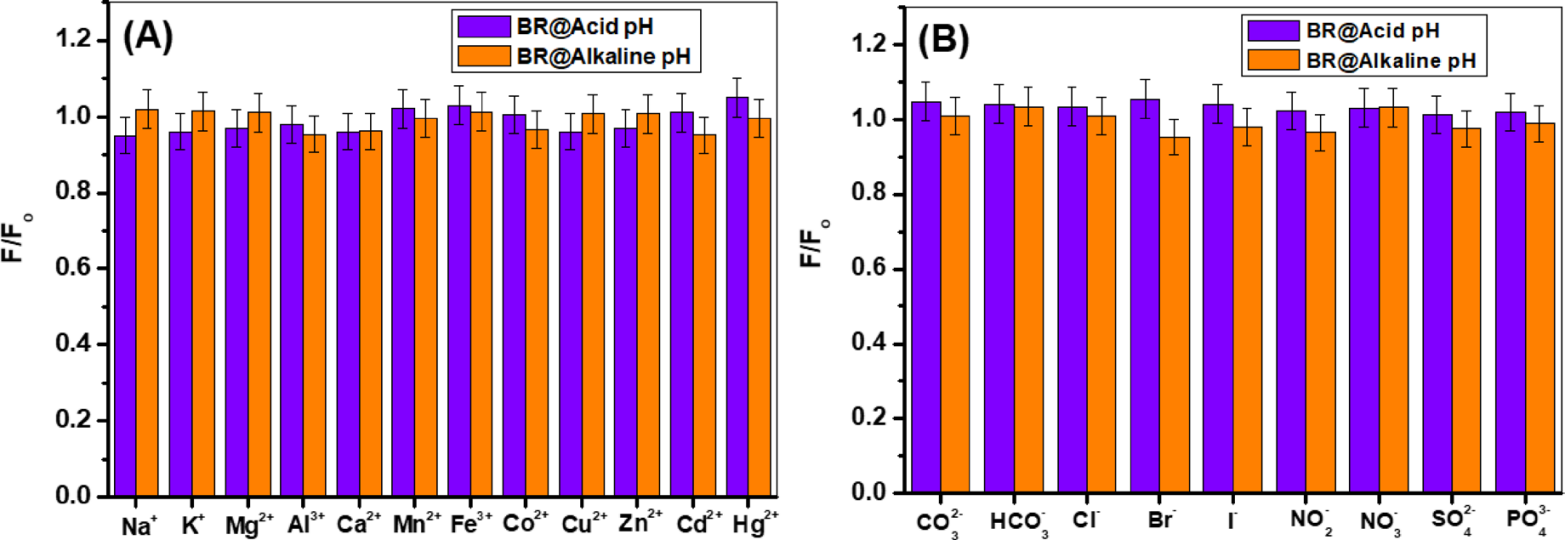
Selectivity of the BR probe towards pH in the presence of co-existing cations (**A**), and Anions (**B**).

The transition of a molecular sensor from a controlled laboratory setting to real-world analytical applications is critically dependent upon its selectivity, a parameter rigorously quantified in Fig. 7. This figure presents a comprehensive interference study evaluating the effects of a variety of biologically and environmentally relevant cations and anions added in 1.0 × 10^−4^ mol L^−1^ concentration that is 10 equivalents to a solution of the BR probe on the fluorescence intensity of the developed fluorophore at its operational acidic and alkaline pH extremes. The metric F/F_0_, where F is the fluorescence intensity in the presence of the potential interferent, and F_0_ is the intensity in its absence, provides a direct measure of signal perturbation. In Fig. 7A, a representative set of common mono-/di-valent ions (Na^+^, K^+^, Mg^2+^, Ca^2+^), multivalent species (Al^3+^, Mn^2+^), and potentially problematic transition/heavy-metal ions (Fe^3+^, Co^2+^, Cu^2+^, Zn^2+^, Cd^2+^, Hg^2+^) are introduced. The fluorescence ratio remains close to unity (F/F₀≈ 1) for both operating acidic and alkaline pH modes. The minimal interference suggests that the proton-binding sites, primarily the phenolate oxygen and the heteroatoms within the rhodanine rings, exhibit a strong thermodynamic preference for H^+^ over these metal cations that do not appreciably create competitive pathways such as metal-assisted quenching, ground-state complex formation, or ion-triggered aggregation that would otherwise distort the pH calibration^44,45^.

Similarly, Fig. 7B confirms that common anions, including CO_3_^2−^, HCO_3_^−^, Cl^−^, Br^−^, I^−^, NO_2_^−^, NO_3_^−^, SO_4_^2−^, and PO_4_^3-^, do not induce detectable fluorescence alterations and show F/F₀ values close to 1 for both acidic and alkaline operation. Mechanistically, this indicates that the sensor’s fluorescence switching is not driven by specific anion recognition (hydrogen bonding, ion pairing, or nucleophilic reactions) but is instead dominated by proton-controlled electronic reorganization. This matters because several of these anions can be chemically “active” in sensing contexts: carbonate/bicarbonate can buffer and locally resist pH changes; halides can modulate ionic strength; nitrite/nitrate and sulfate/phosphate can participate in specific ion pairing or alter solvation microenvironments^45,46^.

The interesting stability in the fluorescence ratio of the developed BR probe against such background chemistry and the selective recognition for only protons and hydroxyl anions, providing robust and anti-interference pH readout validating the probe’s suitability for direct application in real chemically complex environments and industrial samples such as real waters, saline media, and buffered systems without the need for extensive sample pretreatment^46–48^.

### The fluorescence pH-driven ON/OFF mechanism

**Fig. 8:**
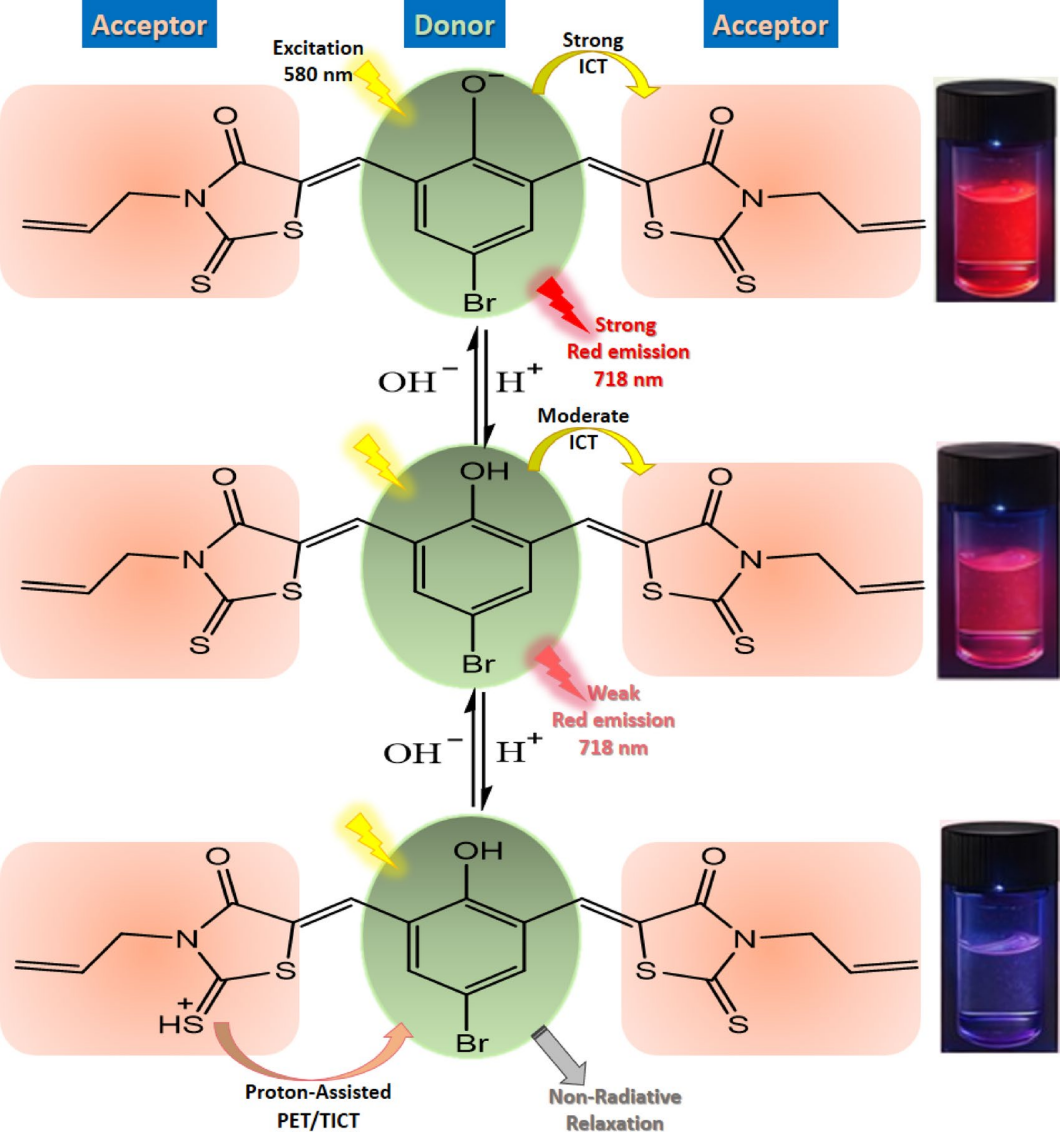
Proposed Scheme for the BR probe acid-base driven protonation/deprotonation equilibria.

The proposed scheme shown in Fig. 8, rationalizes the pH-dependent OFF/ON reversibility by treating the fluorophore as a donor–π–acceptor system whose ground-state protonation pattern pre-sets the excited-state relaxation pathway. Under alkaline conditions (top structure), deprotonation of the central phenolic unit produces a phenolate, which is a substantially stronger electron donor than the neutral phenol. This boosts donor-to-acceptor electronic communication through the conjugated allyl bridges, stabilizes an ICT emissive manifold, and thereby yields the “strong fluorescent” state depicted in the photograph. Such phenol/phenolate switching is a well-established design handle in modern pH probes because it converts a simple Brønsted alteration into a large optical contrast^49,50^.

In the near-neutral regime (middle structure), the dye resides predominantly in the neutral phenolic form; the π-framework remains intact and still supports emission, but the donor strength is lower than in the phenolate state, so the ICT character and therefore the radiative rate are reduced relative to the fully deprotonated form. Upon strong acidification (bottom structure), a protonation at the most basic heteroatom-rich sites is proposed, coupled with restoration of the phenol (–OH). Protonation of acceptor segments commonly disrupts the balanced donor–acceptor resonance that supports efficient ICT and can instead favor fast non-radiative decay, including conformational relaxation into twisted charge-transfer geometries (TICT) or other internal conversion pathways that quench fluorescence^3,51^.

The fluorescence “OFF” state of BR under strong acidification could be proposed via proton-assisted deactivation of the emissive ICT manifold, rather than merely the absence of the phenolate form. When the medium becomes strongly acidic, the phenolic donor is fully protonated (neutral –OH), which reduces donor strength and weakens donor–π–acceptor coupling. In addition, the heteroatom-rich acceptor termini provide plausible secondary protonation sites under very low pH (e.g., thiocarbonyl and/or carbonyl motifs within the rhodanine-like framework). Such protonation can disrupt the resonance balance required for efficient ICT, increase the energetic accessibility of non-radiative pathways, and promote rapid deactivation through proton-assisted PET/TICT-type relaxation and internal conversion, thereby yielding the observed fluorescence quenching (OFF state)^3,51^.

Importantly, the acid-induced quenching occurs with a conserved emission band shape and rapid stabilization to a steady low-intensity plateau, supporting that the OFF state corresponds to a rapidly established protonation-controlled equilibrium rather than slow aggregation or irreversible decomposition. Furthermore, the durable cycling between acidic and alkaline conditions indicates that the protonation steps—whether limited to the phenolic site or accompanied by additional acceptor protonation at extreme acidity that remain reversible under the operational sensing protocol.

### Application of BR probe in pH-assaying in real matrices- accuracy and preciseness

Table 1 provides a practical validation for the BR fluorophore as a spectrofluorometric pH probe across chemically distinct matrices (waters vs. fruit juices), using triplicate measurements (*n* = 3). The results show excellent accuracy, with recoveries within the range (98.9–102.3%), which are accepted limits for quantitative analytical methods, indicating that the pH values estimated from the fluorescence response agree closely with the values taken from the calibrated glass electrode output. Interestingly, the “lake water spiked alkaline pH” entry (10.58 ± 0.12 vs. 10.64; 99.4% recovery) confirms that the method remains reliable in the high-pH operating regime. The precision expressed as %RSD revealed estimates ranging from values 0.93–1.13%, signifying low dispersion among replicates and a stable fluorescence readout. The juice results further highlight matrix tolerance. Fresh lemon and orange juices are inherently colored and compositionally complex (organic acids, sugars, phenolics, and suspended colloids), which can distort fluorescence *via* inner-filter effects^52^. The applied protocol is to filtrate then dilute 3.0 mL of juice to 10.0 mL with deionized water prior to measurement. This is analytically meaningful because it lowers absorbance and turbidity, thereby suppressing inner-filter and improving the fluorescence linearity of fluorescence-based calibration. Even after dilution, the method maintains high accuracy (lemon: 2.67 ± 0.03 vs. 2.61; orange: 3.88 ± 0.04 vs. 3.85) and low %RSD (~ 1.03–1.12%), implying that the remaining matrix components do not significantly perturb the protonation-controlled emissive equilibrium^53,54^.

**Table 1:** Recovery and precision of the BR probe for the spectrofluorometric assay of pH in water and juice samples, *n* = 3.

Sample	pH taken	pH found ± SD	Recovery%	%RSD
Tap Water	6.41	6.47 ± 0.06	100.9	0.93
Lake Water	8.63	8.54 ± 0.08	98.9	0.94
Lake Water spiked alkaline pH	10.64	10.58 ± 0.12	99.4	1.13
Fresh Lemon Juice	2.61	2.67 ± 0.03	102.3	1.12
Fresh Orange Juice	3.85	3.88 ± 0.04	100.8	1.03

A comparison assessment of the current probe’s performance with representive rhodanine-based fluorescent systems are outlined in Table S1. The selected previously published structures could be generally classified as rhodanine-cyanine systems, mono-rhodanines, donor-acceptor fluorophores, and rhodamine-rhodanine hybrids. Regardless of their structural variety, the majority of systems incorporate single-acceptor frameworks or monofunctionalized that limit both the intramolecular charge transfer (ICT) modification and the potential to tune photophysical and pH-responsive properties. For instance, mono-rhodanine Schiff base sensors that desingned by Yang et al.^9^, function mainly in neutral environments and lack pH responsiveness, whereas other mono-rhodanines have a relatively low sensitivity (~ 10^−6^ mol L^−1^) and only work in a narrow pH range (4–10)^55^. Rhodanine–cyanine hybrids almost solve this issue by increasing the sensing ranges beyond acidic to basic environments (pH 1–14), although their practical utility is hindered by limited response kinetics (~ 100 min), which prohibits real-time monitoring^14^. On the other hand, rhodamine–rhodanine hybrid structures have been demonstrated to possess pH-sensitive turn-on fluorescence behaviors. Nevertheless, their sensing range is still narrow (pH 4.2–5.2, pKa ≈ 4.85), which limits their ability to work in many different environments^15^. 2-Triphenylamine-1,3-dia[2-(3-ethyl-4-oxo-thiazolidin-2-ylidene)-malononitrile] (2RDNTPA) fluorophores have enhanced photophysical characteristics and are frequently utilized in biological imaging; nonetheless, they remaining face challenges from restricted structural tunability and are not suitable for wide-range pH detection applications^26^. Conversely, the current study proposes a *bis*-allyl rhodanine structure with a dual acceptor system, which permits greatly increased intramolecular charge transfer (ICT). This structural design results in deep-red emission at 718 nm, a significant Stokes shift of 149 nm, and a distinct reversible OFF/ON fluorescence response over an extensive pH range from 1.0 to 12.0, with a pKa of around 5.2. Furthermore, this system also responds rapidly (~ 30 s), exceeding previously documented systems in both spectral and kinetic performances. In summary, this finding unambiguously indicate that the current probe, rather than a progressive modification of traditional systems, offers a significant advancement in rhodanine-based fluorophore design *via* dual-acceptor molecular building. When comparing red-emitting fluorescent probes that are not based on a rhodanine framework^56–59^, as displayed in Table 2, previously published pH fluorescent sensors typically demonstrate emission maxima within 438–660 nm and relatively small Stokes shifts (13–87 nm), which may hinder detection efficacy owing to spectral overlap and self-absorption. On the other hand, the BR probe shows an unusually large Stokes shift of 149 nm along with a prominent red emission at 718 nm (Table 2). This value is a major photophysical benefit of the current design and is much higher than those of reported systems. Further demonstrating BR’s suitability for sensitive pH sensing is its competitive analytical performance in terms of pKa (~ 5.2) and limit of detection (LOD = 0.21 in the acidic range and 0.32 in the basic range). Collectively, these results confirm that the BR system is a well-designed rhodanine-based platform with enhanced spectral separation and balanced sensing performance when compared to previously published red-emitting pH probes.

**Table 2:** Comparison of reported red-emitting fluorescent pH probes and the *bis*-rhodanine D–π–A system.

Probe	λ_em_ (nm)	Stokes shift (nm)	pK_a_	LOD	Synthesis method	Ref
4-(Pentafluorophenyl)-5-aryl-2*H*-imidazoles	438	87	–	0.2	Suzuki-Miyaura functionalization strategy	56
Diazaoxatriangulenium Dyes	585	32	5.38	0.22	Nanoprecipitation method	57
(3-Ethyl-2,4-dimethyl)-BODIPY	553	13	5.13	–	TFA-catalyzed pyrrole–aldehyde condensation	58
*N*,*N*′-(2-ethylhexyl)-1,13- dimethoxyquinacridinium doped polystyrene nanoparticles	660	–	6.33	–	Emulsification method	59
Bis-rhodanine D–π–A	718	149	5.2	0.21 (Acid range) 0.32 (Basic range)	US/Green DES	This work

## Conclusions

In summary, an ultrasound-assisted DES-mediated protocol enabled the selective synthesis of a *bis*-rhodanine derivative (BR) under mild conditions, delivering excellent *Z*-selectivity and a high isolated yield of 96%. The structure of the obtained product was unambiguously established through comprehensive spectroscopic and analytical characterization. The designed BR functions as a robust red-emitting pH probe with two analytically useful regimes: fluorescence quenching under strong acidification and fluorescence sensitization under alkalinity. The reversibility between the fluorescence OFF mode at pH 2.0 and the fluorescence ON mode at pH 10.5 is mechanistically consistent with a rapid deprotonation/protonation equilibrium that modulates donor strength and charge-transfer stabilization. Under alkaline conditions, the deprotonation of the phenolic donor generates a phenolate, substantially increasing the donor strength and strengthening the donor–π–acceptor interaction, thereby enhancing the push–pull coupling across the conjugated bridge. This state is proposed to stabilizes an intramolecular charge-transfer (ICT) excited configuration with enhanced oscillator strength and a comparatively efficient radiative decay channel, producing the observed fluorescence “ON” response. As the medium becomes acidic, reprotonation suppresses donor ability and can additionally promote protonation at heteroatom-rich acceptor sites, weakening ICT stabilization and increasing the energetic accessibility of competing deactivation routes. In this protonated regime, nonradiative pathways such as internal conversion facilitated by stronger vibronic coupling, conformational relaxation toward a less emissive charge-transfer geometry, and/or protonation-enabled electron/charge redistribution that accelerates excited-state quenching, leading to fluorescence “OFF”. Upon acidification, reprotonation (and possible protonation of heteroatom-rich acceptor sites) weakens ICT stabilization and increases the competitiveness of non-radiative channels, such as internal conversion and conformationally assisted charge-transfer deactivation, yielding the “Fluorescence OFF” state. The probe provides linear calibration in both acidic (pH 6.5–1.0) and alkaline (8.5–12.0) solutions, responds rapidly, and sustains repeated switching without significant signal loss. Therefore, BR interestingly demonstrates dual-regime pH sensing: a low-pH fluorescence OFF state and a high-pH fluorescence ON state together with a well-defined near-neutral transition (pH 6.5–8.5), thereby supporting its use as a broadly applicable probe without overstating uniform sensitivity across the entire pH span. Minimal interference from common ions and near-quantitative recoveries in water and diluted juice samples support the practical applicability of this method for routine pH assessment in complex matrices with high accuracy and precision.

### Limitations and recommendations for future work

This work demonstrates a reliable and sustainable *bis*-rhodanine fluorophore for wide-range optical pH sensing with high sensitivity, reversibility, and validated real-sample performance. The study fully addresses its objectives within aqueous systems relevant to environmental and food analysis. Future studies may extend this platform to more complex matrices, longer-term operation, and continuous monitoring. Additionally, integration into solid-state or portable formats offers promising opportunities for practical pH sensing applications. Complementary DFT or time-resolved studies will be valuable in future work to provide definitive validation of the ICT assignment. Accordingly, lifetime determination will be pursued in future work using appropriate nanosecond-resolved techniques, which would further strengthen mechanistic interpretation and provide an additional quantitative benchmark for probe performance.

## Supplementary Information

Below is the link to the electronic supplementary material.

Supplementary Material 1

## Data Availability

All data generated or analyzed during this study are included in this published article.
