# Supplementary material for: An innovative bis-allyl rhodanine robust red fluorophore for wide-range optical pH sensing with reversible and durable acidic–alkaline OFF/ON fluorescence
Source: Sci Rep. 2026 Jul 10;16:21508. doi: 10.1038/s41598-026-51070-4 (PMC13350916; doi:10.1038/s41598-026-51070-4)
Supplement: Supplementary file 1 — Supplementary Material 1 [file 41598_2026_51070_MOESM1_ESM.pdf]

Electronic Supporting Information

An Innovative Bis-Allyl Rhodanine Robust Red Fluorophore for Wide-Range Optical pH Sensing with Reversible and Durable Acidic–Alkaline OFF/ON Fluorescence

Wael A. A. Arafa,[1]* AbdElAziz A. Nayl,[1] Abd El-Naby I. Essawy,[2] Stefan Bräse,[3]* and Amr A. Essawy[1]

[1]Department of Chemistry, College of Science, Jouf University, 72341, Sakaka, Al Jouf, Kingdom of Saudi Arabia

[2]Department of Chemistry, Faculty of Science, Fayoum University, 63514, Fayoum, Egypt

[3]Institute of Biological and Chemical Systems-Functional Molecular Systems (IBCS-FMS), Kaiserstrasse 12, 76131, Karlsruhe, Germany

*Corresponding. waarafa@ju.edu.sa; stefan.braese@kit.edu

Table of Contents

No.	Content	Page No.
1.	Instrumentation and materials	S1
2.	Synthesis of 5-bromo-2-hydroxyisophthalaldehyde (**2**)	S2
3.	Synthesis of (5Z,5'Z)-5,5'-((5-bromo-2-hydroxy-1,3-phenylene)bis(methanylylidene))bis(3-allyl-2-thioxothiazolidin-4-one) (**4**)	S2
4.	Copies of NMR and HRMS	S3
5.	Fig. S6. Recyclability of the glycerol/proline DES over four consecutive cycles	S7
6.	Fig. S7. UV–Vis absorption spectra of the developed BR fluorophore under UV illumination for 60 min. (A) and (B) Pseudo–first-order kinetic plots (ln(A/A_0) vs. time), ([BR] = 1 x 10-5 mol. L-1)	S7
7.	Fig. S8. Variation of the fluorescence spectrum of the **BR** pH probe ([**BR**] = 5.0 μM) upon increasing alkalinity from pH 6.5 to 8.5. "Fluorescence sensitization" (A); the linear regression equation and the developed calibration curve (B)	S8
8.	Table S1. Comparative structural design and sensing performance of rhodanine-based fluorescent probes reported in the literature and the present work.	S8
9.	References	S9

Instrumentation and Materials

FTIR and UV-Vis spectra were recorded on Shimadzu IR-Tracer 100 (Kyoto, Japan) and an Agilent spectrophotometer (Agilent Technologies, Santa Clara, CA, USA), respectively. $^{1}H/^{13}C$ NMR spectra were obtained on a Jeol 600 MHz spectrometer

(JEOL, Peabody, MA, USA) in DMSO-d_6. All reagents were used as received from Sigma-Aldrich (St. Louis, MO, USA)

Synthesis of 5-bromo-2-hydroxyisophthalaldehyde (2) [1]

A mixture of 4-bromophenol (**1**, 1.0 mmol) and hexamethylenetetramine (8.0 mmol) in anhydrous trifluoroacetic acid (25.0 mL) was heated at 110 ^{o}C for 48 h, affording yellow solution. Upon cooling to room temperature, the reaction mixture was added to aqueous solution of HCl (4 M, 50.0 mL) and stirred for 5 h. The precipitated solid was collected by filtration and washing with water (3 x 15.0 mL) to yield the desired product as yellow crystals (93%). ^{1}H NMR (600 MHz, acetone-d_6): δ = 8.19 (s, 2H, Ar-H; 4H&6H), 10.28 (s, 2H, 2C*H*O), 11.71 ppm (s, 1H, O*H*). ^{13}C NMR (150 MHz, acetone-d_6): δ = 112.45 (Ar-*C5*), 126.04 (Ar-*C*1,3), 140.47 (Ar-*C4,6*), 192.54 ppm (*C*=O). HRMS (ESI) calcd for $C_8H_5BrNaO_3$ [M + Na$^+$]$^+$: 250.9314; found: 250.9310.

Synthesis of (5Z,5'Z)-5,5'-((5-bromo-2-hydroxy-1,3-phenylene)*bis*(methanylylidene))*bis*(3-allyl-2-thioxothiazolidin-4-one) (4)

A mixture of 5-bromo-2-hydroxyisophthalaldehyde (**2**, 1.0 mmol) and 3-allyl-2-thioxothiazolidin-4-one (**3**, 2.0 mmol) in glycerol/proline (2:1, 5.0 g) was sonicated at 80 ^{o}C for 90 min as monitored by TLC. Upon addition of water (20.0 mL), a solid precipitated and was collected by filtration, then washed with ethanol (3x10.0 mL) to afford compound **4** in 96% yield without further purification. The recyclability of the DES (glycerol/proline, 2:1) was evaluated over multiple reaction cycles. After completion of the reaction, water was added to precipitate the product, and the DES phase was recovered from the filtrate. The recovered DES was reused directly after removal of excess water under reduced pressure. The results demonstrated consistent performance over four successive cycles. ^{1}H NMR (600 MHz, DMSO-d_6): δ = 4.59 (br, CH_2=CH-CH_2-, 4H), 5.02-5.11 (m, CH_2=CH-CH_2-, 4H), 5.78-5.84 (m, CH_2=C*H*-CH_2-, 2H), 7.79 (s, 2H, Ar-H; 4H&6H), 8.21 (s, 2H, =C*H*), 11.60 ppm (s, 1H, O*H*). ^{13}C NMR (150 MHz, DMSO-d_6): δ = 47.93 (CH_2=CH-*C*H_2), 117.08 (*C*H_2=CH-CH_2-), 134.05 (CH_2=*C*H-CH_2), 115.55 (Ar-*C5*), 116.07 (Thiazole-*C*5), 119.14 (Ar-*C1,3*), 129.98 (Ar-*C4,6*), 145.66 (=*C*H), 156.97 (Ar-*C2*), 166.63 (*C*=O), 194.36 ppm (*C*=S). HRMS (ESI, Positive ion mode) calcd for $C_{20}H_{15}BrN_2O_3S_4$ [M]$^+$: 537.9149; found: 537.9197. Anal. calcd for $C_{20}H_{15}BrN_2O_3S_4$: C, 44.53; H, 2.80; N, 5.19%. Found: C, 44.59; H, 2.77; N, 5.13%.

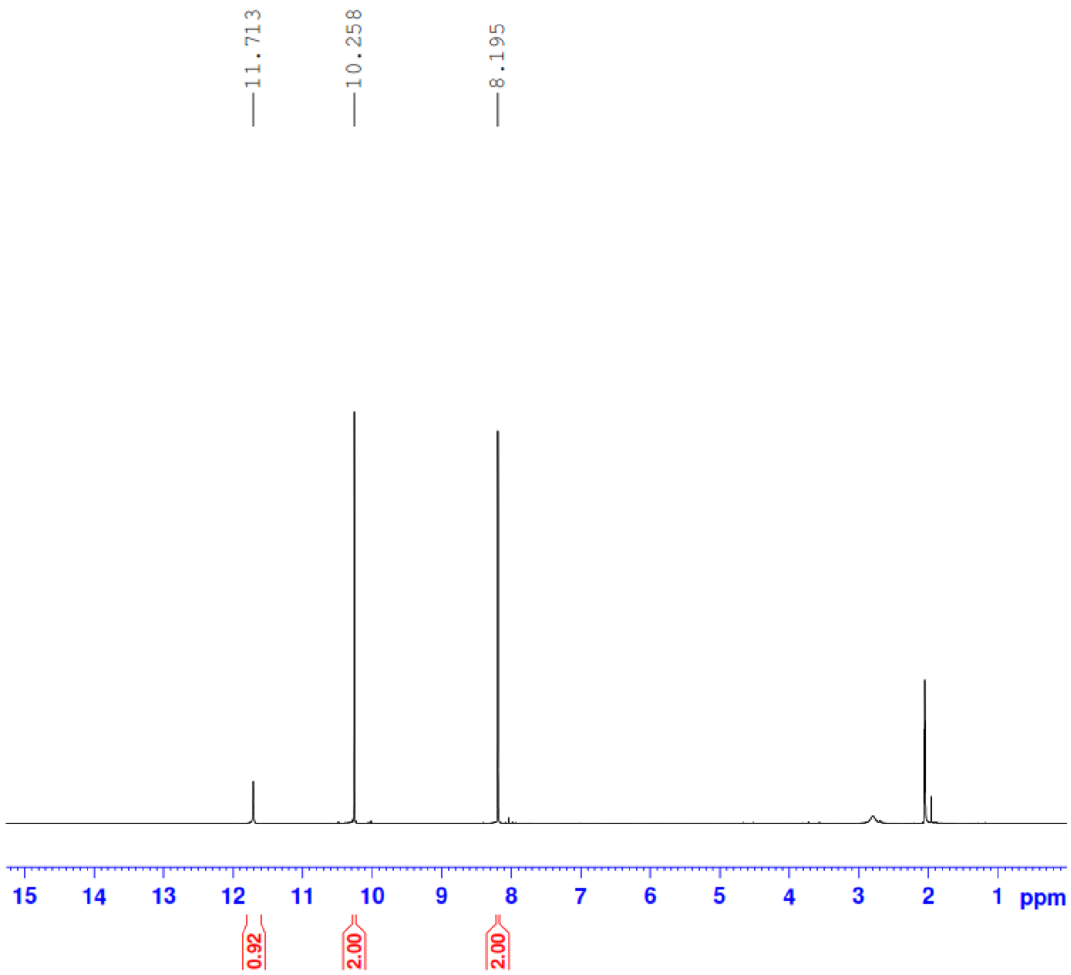

Fig. S1. ^{1}H NMR (Acetone-*d6*) of (**2**)

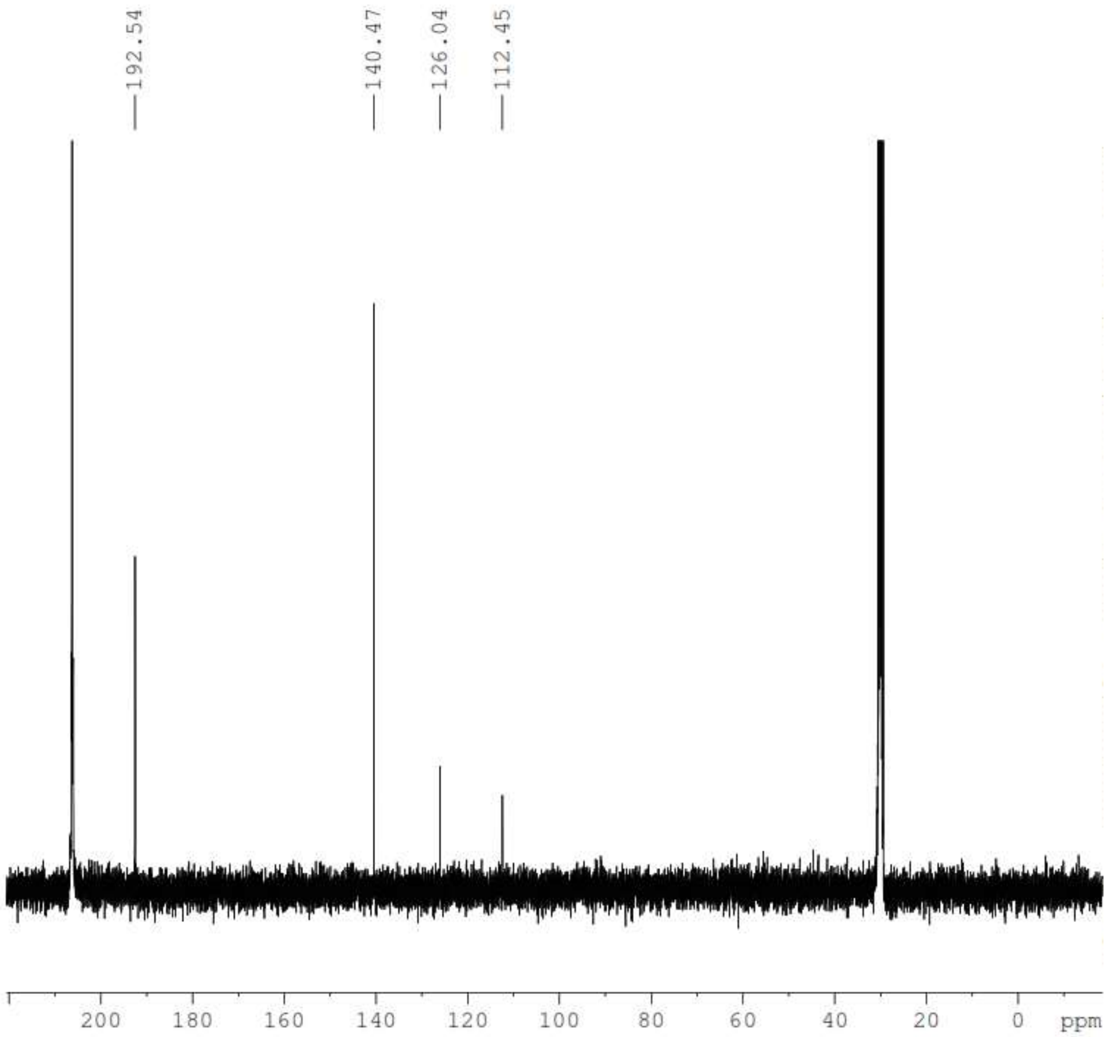

Fig. S2. ^{13}C NMR (Acetone-*d6*) of (**2**)

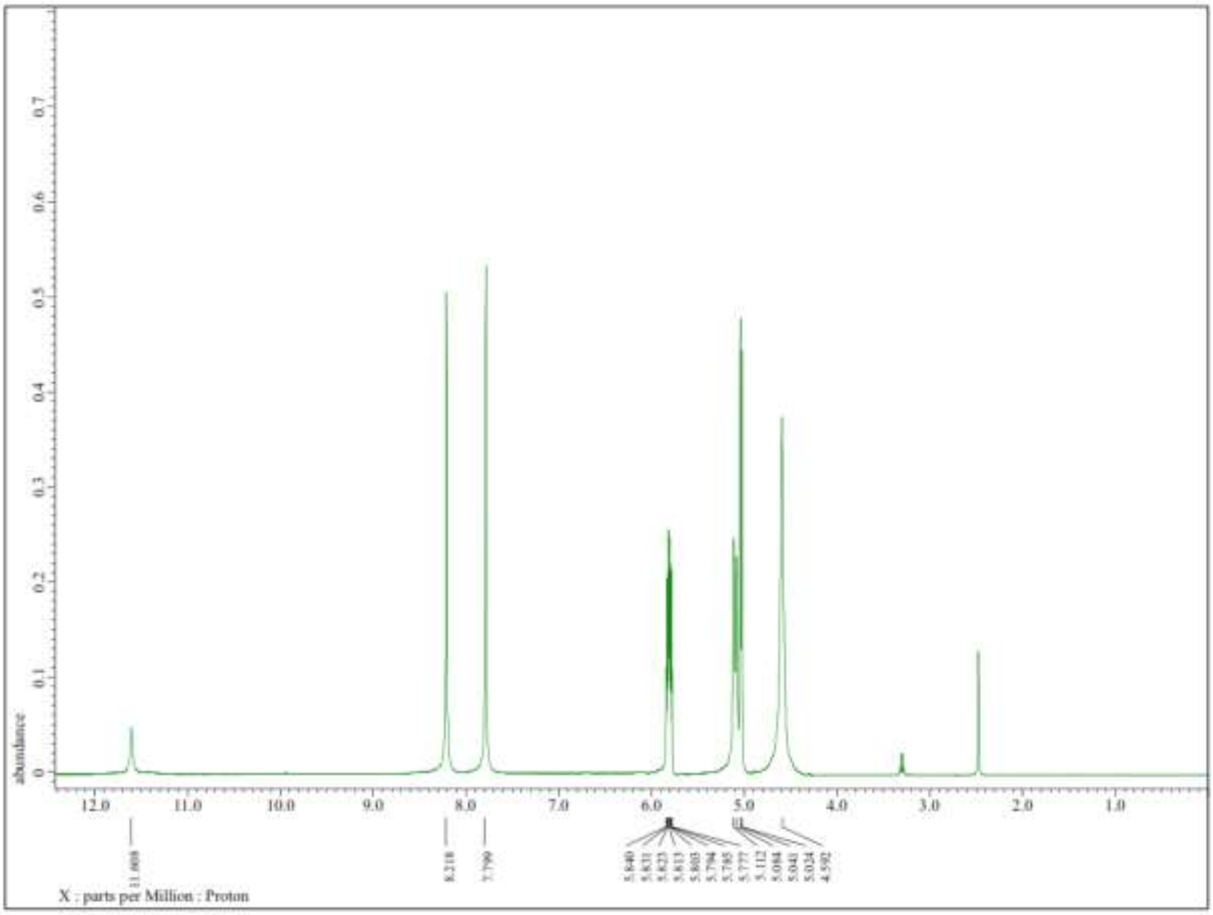

Fig. S3. ^{1}H NMR (DMSO-*d6*) of (**4**)

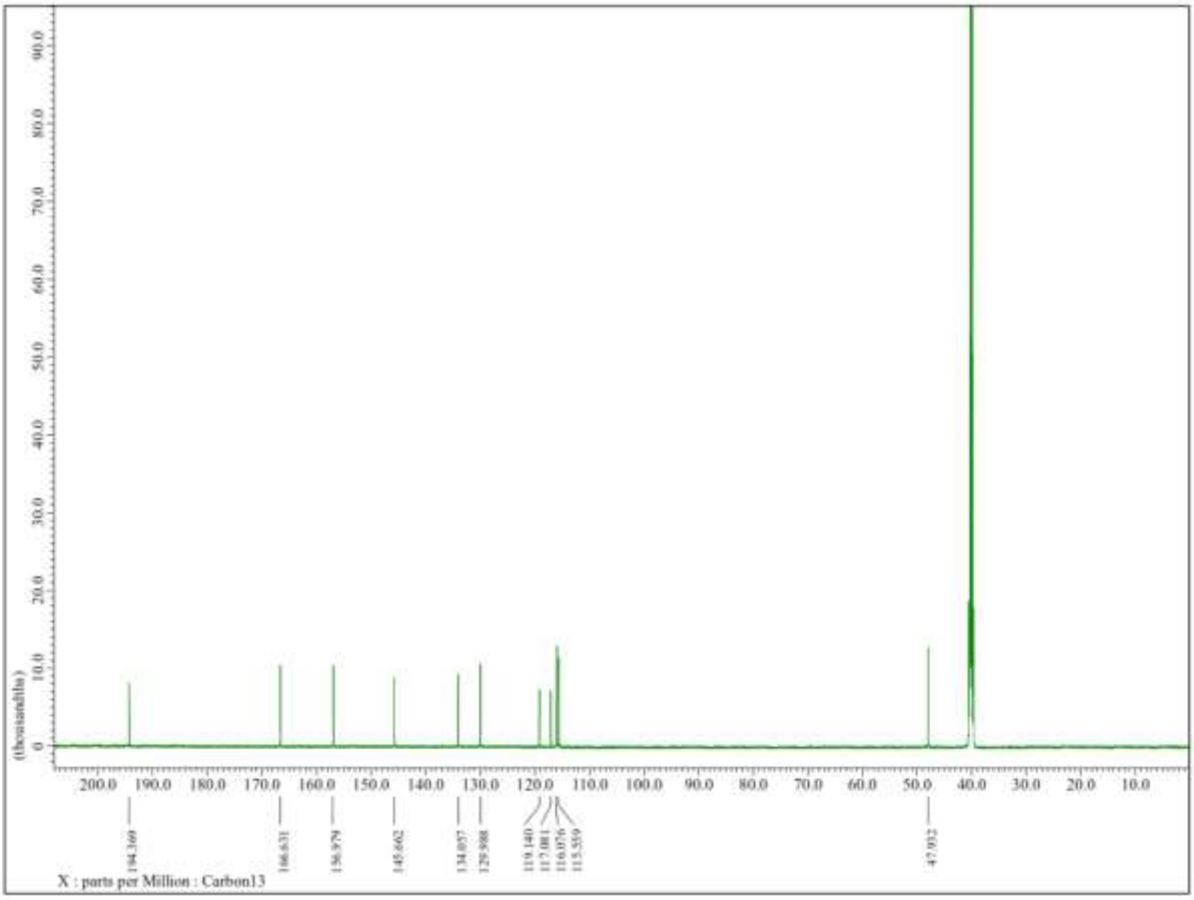

Fig. S4. ^{13}C NMR (DMSO-*d6*) of (**4**)

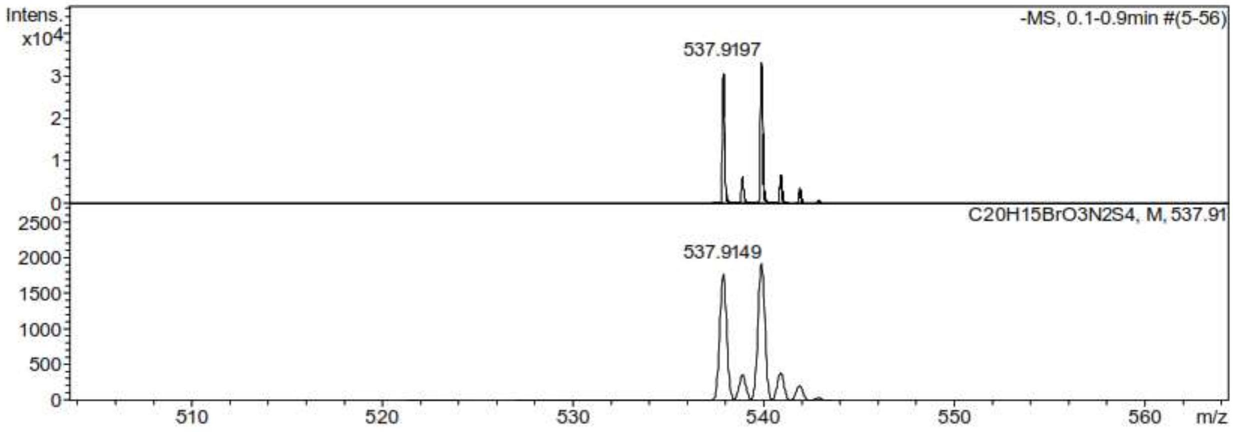

Fig. S5. HRMS of (**4**)

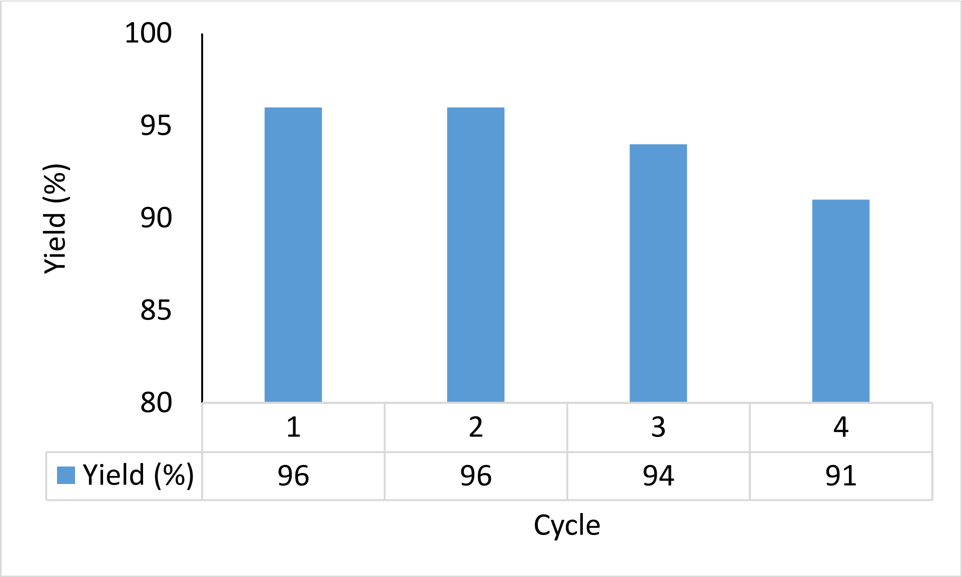

Fig. S6. Recyclability of the glycerol/proline DES over four consecutive cycles

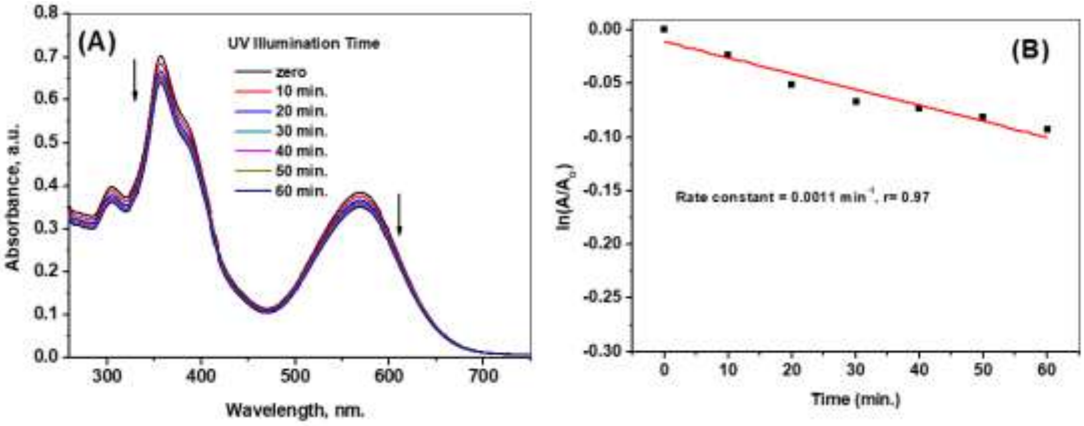

Fig. S7. UV–Vis absorption spectra of the developed **BR** fluorophore under UV illumination for 60 min. (A) and (B) Pseudo–first-order kinetic plots ($\ln(A/A_0)$ *vs.* time), ([**BR**] = 1 x 10^{-5} mol. L^{-1})

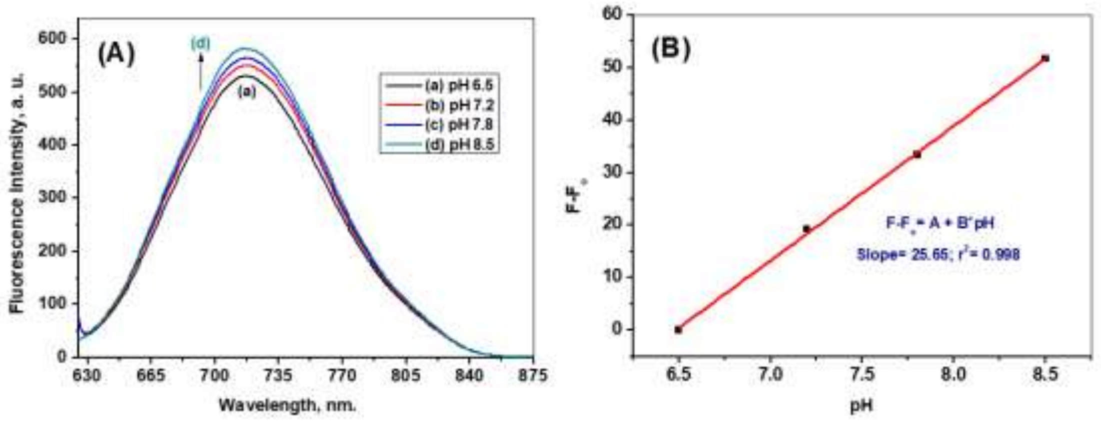

Fig. S8. Variation of the fluorescence spectrum of the **BR** pH probe ([**BR**] = 5.0 µM) upon increasing alkalinity from pH 6.5 to 8.5. "Fluorescence sensitization" (A); the linear regression equation and the developed calibration curve (B).

Table S1. Comparative structural design and sensing performance of rhodanine-based fluorescent probes reported in the literature and the present work.

Probe (Ref.)	Structural Feature	pKa	Emission λ (nm)	Application/ Range	Response Type	Key Limitation
[2]	Benzylidene–rhodanine Schiff base	pKa ≈ 7	500–550	In^{3+} sensing	Turn-on	Not pH-responsive; mono-functionalized
[3]	Benzylidene–rhodanine	–	~570	Cu^{2+} sensing with pH range (1–14)	Turn-on	Not pH-responsive; mono-functionalized
[4]	Rhodamine–rhodamine hybrid	pKa = 4.85	~580–600	Narrow acidic range (4.2–5.2)	Turn-on	Very narrow pH window
[5]	Cyanine-rhodanine hybrid	pKa ≈ 5.0	~583	Wide pH (1–14)	Colorimetric and fluorescent	Slow response (~100 min)
[6]	2RDNTPA* (rhodanine–malononitrile)	–	629	Bioimaging fluorophore	Intensity-based	Limited tunability; not designed for wide-range pH
This work	*Bis*-allyl D–π–A rhodanine (dual acceptor system)	pKa ≈ 5.2	718	Acid: 1.0–6.5 Base: 8.5–12.0	Reversible OFF/ON fluorescence	–

*2RDNTPA = 2-Triphenylamine-1,3-dia[2-(3-ethyl-4-oxo-thiazolidin-2-ylidene)-malononitrile]

References

1. Wael A. A. Arafa, Markus D. Kärkäs, Bao-Lin Lee, Torbjörn Åkermark, Rong-Zhen Liao, Hans-Martin Berends, Johannes Messinger, Per E. M. Siegbahna and Björn Åkermark, Dinuclear manganese complexes for water oxidation: evaluation of electronic effects and catalytic activity, *Phys. Chem. Chem. Phys.*, **2014**, *16*, 11950-11964. https://doi.org/10.1039/C3CP54800G
2. M. Yang, J.J. Lee, D. Yun, H. So, Y. Yi, M.H. Lim, H. Lee, K.-T. Kim, C. Kim, *In vitro* and *vivo* application of a rhodanine-based fluorescence sensor for detection and bioimaging of In^{3+} at neutral pH. *J. Photochem. Photobiol. A Chem.*, **2023**, *434*, 114249. https://doi.org/10.1016/j.jphotochem.2022.114249
3. E. Kalay, O. Özbek, M. Elik, C. Berkel, O.N. Aslan, The synthesis, sensor and biological properties of two novel rhodanine derivative molecules. *J. Indian Chem. Soc.*, **2025**, *102*, 101591. https://doi.org/10.1016/j.jics.2025.101591
4. X.-X. Zhaoa, X.-P. Chenb, S.-L. Shena, D.-P. Lia, S. Zhoua, Z.-Q. Zhoua, Y.-H. Xiaoa, G. Xia, J.-Y. Miaob, B.-X. Zhaoa, A novel pH probe based on rhodamine-rhodanine platform, *RSC Adv.*, **2014**, *4*, 50318. https://doi.org/10.1039/C4RA07555B
5. Y. Li, Y. Qi, Z. Xu, Y. Song, H. Li, J. Zhang, J. Tang, J. Zhou, A cyanine-based dual-modal probe for fluorimetric detection of mercury ion and colorimetric sensing of pH. *Color. Technol.*, **2022**, *138*, 427. https://doi.org/10.1111/cote.12603

6. Z. Li, B. Huang, G. Xing, Y. Wang, W. Yuan, Y. Wu, R. Yu, T. Zou, Y. Tao, Design, synthesis and application in biological imaging of a novel red fluorescent dye based on a rhodanine derivative. *RSC Adv.*, **2021**, *11*, 160. https://doi.org/10.1039/D0RA08998B
